# Excited States and Optical Properties of Hydrogen-Passivated Rectangular Graphenes: A Computational Study

**DOI:** 10.1038/s41598-019-44258-4

**Published:** 2019-05-28

**Authors:** Deepak Kumar Rai, Alok Shukla

**Affiliations:** Department of Physics, Indian Institute of Technology Bombay, Powai, Mumbai, 400076 India

**Keywords:** Computational chemistry, Chemical physics

## Abstract

In this paper, we perform large-scale electron-correlated calculations of optoelectronic properties of rectangular graphene-like polycyclic aromatic hydrocarbon molecules. Theoretical methodology employed in this work is based upon Pariser-Parr-Pople (PPP) *π*-electron model Hamiltonian, which includes long-range electron-electron interactions. Electron-correlation effects were incorporated using multi-reference singles-doubles configurationinteraction (MRSDCI) method, and the ground and excited state wave functions thus obtained were employed to calculate the linear optical absorption spectra of these molecules, within the electric-dipole approximation. As far as the ground state wave functions of these molecules are concerned, we find that with the increasing size, they develop a strong diradical open-shell character. Our results on optical absorption spectra are in very good agreement with the available experimental results, outlining the importance of electron-correlation effects in accurate description of the excited states. In addition to the optical gap, spin gap of each molecule was also computed using the same methodology. Calculated spin gaps exhibit a decreasing trend with the increasing sizes of the molecules, suggesting that the infinite graphene has a vanishing spin gap.

## Introduction

Despite many attractive properties, graphene has still not found applications in opto-electronic devices, because of the lack of a band gap. Therefore, in recent times, considerable amount of research effort has been directed towards graphene nanostructures such as quantum dots^1–6^, and nanoribbons^7^, which are expected to have band gaps because of quantum confinement. The idealized graphene nanoribbons (GNRs) have either zigzag or armchair edges, with substantially different electronic structure, and related properties. Theoretical studies reveal that zigzag GNRs (ZGNRs) exhibit edge magnetism with possible applications in spintronic devices^8,9^, while the armchair GNRs (AGNRs) are direct bandgap semiconductors, with potential optoelectronic applications^10,11^. If we consider either an AGNR or a ZGNR of a given width, and hypothetically cut it at two places perpendicular to its width the resultant rectangular structure, referred to as a rectangular graphene molecule (RGM), will have both armchair and zigzag type edges. We also assume that the edge carbon atoms of RGMs are passivated by H atoms, so as to neutralize the dangling bonds, thus preventing edge reconstruction, and allowing them to retain their symmetric shapes. Such structures, obviously, will be nothing but polycyclic aromatic hydrocarbon molecules. In this work we perform a computational study of optoelectronic properties of RGMs, with the aim of understanding as to how they are influenced by the edge structure. Because the electronic properties of ZGNRs and AGNRs are very different from each other, it is of considerable interest as to how the electronic properties of RGMs, which have both zigzag and armchair edges, evolve with the edge lengths. Such an understanding will help us in tuning the optoelectronic properties of RGMs by manipulating their edges.

In our theoretical approach we consider RGMs to be systems whose low-lying excited states are determined exclusively by their *π* electrons, with negligible influence of *σ* electrons. As a result we adopt a computational approach employing the Pariser-Parr-Pople (PPP) *π*–electron Hamiltonian^12,13^, and the configuration interaction (CI) method, used in several of our earlier works on conjugated polymers^14–20^, polycyclic aromatic hydrocarbons^21,22^, and graphene quantum dots^23–25^. We adopt this approach to study RGMs with the number of carbon atoms ranging from 28 to 56, corresponding to structures with increasing edge lengths in both armchair, and zigzag, directions. Adopting the notation that RGM-*n* denotes a rectangular graphene structure with *n* carbon atoms, the chemical analogs of RGM-28, -30, -36, -40, -42, -50, and -56 are aromatic compounds bisanthenes, terrylene, tetrabenzocoronene, quaterrylene, teranthene, pentarylene and quateranthene, respectively. On comparing our theoretical results to the measured ones on these molecules, we obtain excellent agreement, thus validating our methodology.

Additionally, using the same MRSDCI methodology, we computed the spin gap of each RGM studied in this work. We find that with the increasing sizes of the RGMs, their spin gaps are decreasing, suggesting that the spin gap of infinite graphene vanishes.

## Structure and Symmetry

**Figure 1:**
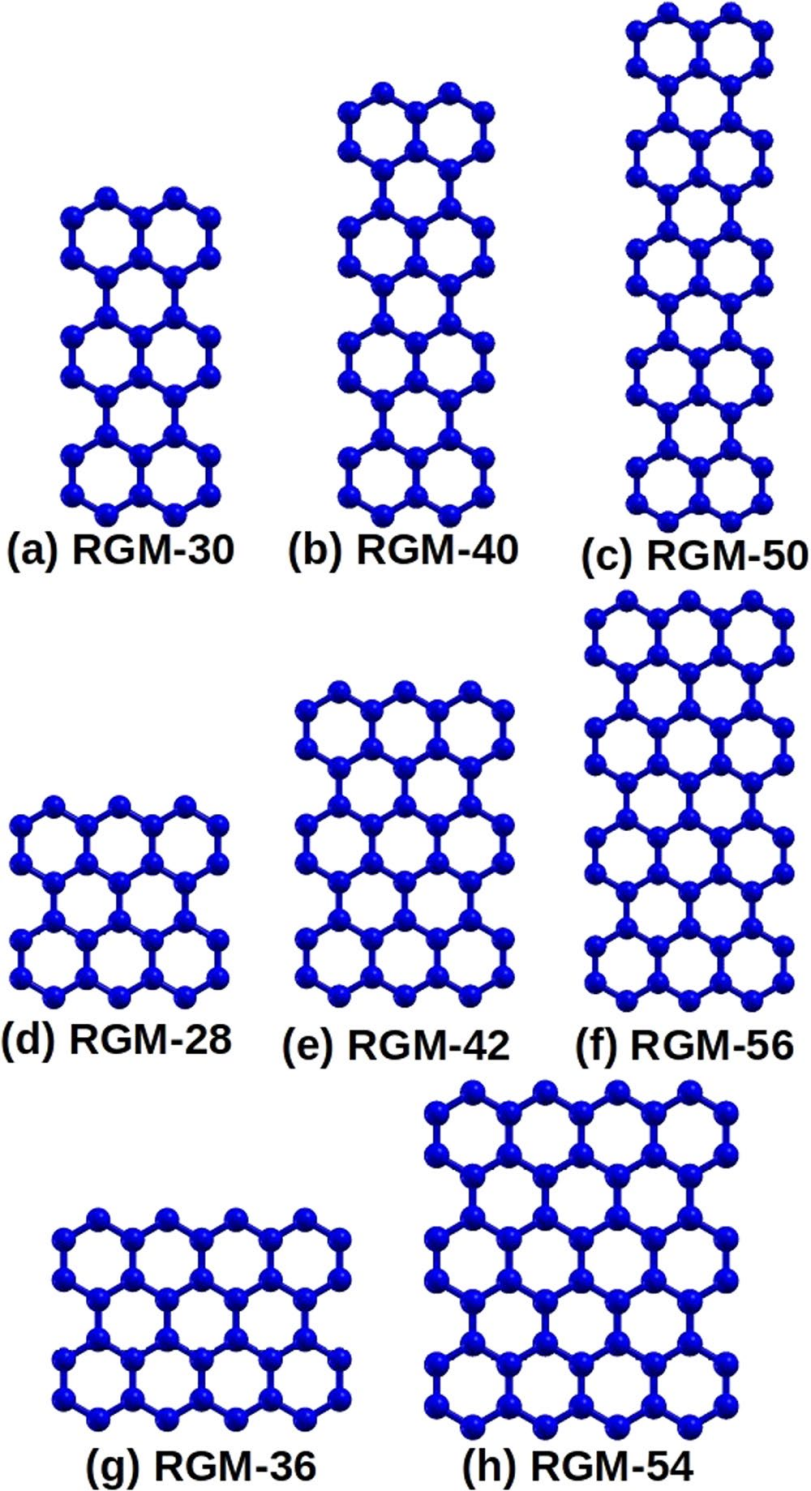
Schematic diagrams of RGMs considered in this work. For all the molecules, edge carbon atoms are assumed passivated by hydrogens. Notation RGM-*n* denotes a rectangular-shaped graphene-like molecule with *n* carbon atoms.

The schematic diagrams of RGMs considered in this work are shown in Fig. 1. As mentioned earlier, we have assumed that the dangling bonds on the edges of the molecules have been saturated by hydrogen atoms. Thus, the molecules considered here can be treated as planar hydrocarbons, exhibiting *π* conjugation. We have assumed that all the RGMs lie in the *xy* plane, with idealized bond lengths of 1.40 Å, and bond angles of 120°.

Within the PPP model based theoretical methodology adopted here, small variations in bond lengths and angles do not make any significant differences to the calculated optical properties of such structures, as demonstrated by us earlier^18,19^. Having assumed the idealized geometry for the RGMs, their point group symmetry is *D*_2*h*_, as in case of polyacenes studied by us earlier^17–20^. Because all the systems considered here have an even number of electrons, their ground state is of ^1^*A*_*g*_ symmetry, so that their one-photon dipole-connected excited states will be of the symmetries: (a) ^1^*B*_3*u*_, accessible by absorbing an *x*-polarized photon, and (b) ^1^*B*_2*u*_, reached through the absorption of a *y*-polarized photon.

## Theoretical Methods

As discussed in the previous section, with hydrogen passivated edges, the molecules considered here are *π*-conjugated systems, and, therefore, in this work we employed Pariser-Parr-Pople (PPP) effective *π*-electron model Hamiltonian^12,13^,

1 $$\begin{array}{c}H=-\sum _{i,j,\sigma }\,{t}_{ij}({c}_{i\sigma }^{\dagger }{c}_{j\sigma }+{c}_{j\sigma }^{\dagger }{c}_{i\sigma })+U\sum _{i}\,{n}_{i\uparrow }{n}_{i\downarrow }\\ \,\,+\sum _{i < j}\,{V}_{ij}({n}_{i}-\mathrm{1)(}{n}_{j}-\mathrm{1),}\end{array}$$

where ${c}_{i\sigma }^{\dagger }({c}_{i\sigma })$ are creation (annihilation) operators for the *p*_*z*_ orbital of spin *σ*, located on the *i*-th carbon atom, while the total number of electrons on the atom is indicated by the number operator ${n}_{i}={\sum }_{\sigma }\,{c}_{i\sigma }^{\dagger }{c}_{i\sigma }$. The first term in Eq. (1) denotes the one-electron hopping processes connecting *i*-th and *j*-th atoms, quantified by matrix elements *t*_*ij*_. We assume that the hopping connects only the nearest-neighbor carbon atoms, with the value *t*_0_ = 2.4 eV, consistent with our earlier calculations on conjugated polymers^14–20^, polyaromatic hydrocarbons^21,22^, and graphene quantum dots^24,25^. The next two terms in Eq. (1) represent the electron-electron repulsion interactions: (a) parameter *U* denotes the on-site term, while (b) *V*_*ij*_ denotes the long-range Coulomb term. The distance-dependence of parameters *V*_*ij*_ is assumed as per Ohno relationship^26^

2 $${V}_{ij}=U/{\kappa }_{i,j}{\mathrm{(1}+0.6117{R}_{i,j}^{2})}^{12},$$

where *κ*_*i*,*j*_ is the dielectric constant of the system, included to take into account the screening effects, and *R*_*i*,*j*_ is the distance (in Å) between the carbon atoms involved. In the present set of calculations we have used two sets of Coulomb parameters: (a) the “screened parameters”^27^ with *U* = 8.0 eV, *κ*_*i*,*j*_ = 2.0(*i* ≠ *j*), and *κ*_*i*,*i*_ = 1.0, and (b) the “standard parameters” with *U* = 11.13 eV and *κ*_*i*,*j*_ = 1.0.

We initiate the computations by performing restricted Hartree-Fock (RHF) calculations for the closed-shell singlet ground states of the RGMs considered here, by employing the PPP Hamiltonian (Eq. (1)), using a computer program developed in our group^28^. The molecular orbitals (MOs) obtained from the RHF calculations are used to transform the PPP Hamiltonian from the site basis, to the MO basis, for the purpose of performing many-body calculations using the CI approach. The correlated-electron multi-reference singles-doubles configuration interaction (MRSDCI) approach was employed in this work, which consists of a CI expansion obtained by exciting up to two electrons, from a chosen list of reference configurations, to the unoccupied MOs^29,30^. The reference configurations included in the MRSDCI method depend upon the targeted states, which, in the present calculations are configurations of symmetry *A*_*g*_ for calculating the ground state, and configurations of *B*_2*u*_ and *B*_3*u*_ symmetries for computing the one-photon excited state wave functions. The MRSDCI calculations are initiated using a single configuration, such as the closed-shell RHF state, as the reference for the ground state, or a suitable set of excited configurations of appropriate point-group symmetries, and singlet spin multiplicity, for representing optically excited states of the system. After performing the MRSDCI calculations with this initial set of reference configurations, the optical absorption spectrum is computed, and those excited excited states are identified which contribute to peaks in it. Next, the wave functions of both ground and excited states are carefully examined, and those configurations are identified, magnitudes of whose coefficients are above a chosen convergence threshold. The next MRSDCI calculation is carried out with an enhanced reference set, obtained by including these additional configurations. This procedure is iterated until the desired physical quantities of the system, such as the excitation energies, optical absorption spectra etc., converge. The lowest eigenvalue and eigenvector obtained from the MRSDCI calculations on the *A*_*g*_ symmetry manifold corresponds to the ground state, while all other states are identified with various excited states. For computing the 1^3^*B*_2*u*_ state needed for calculating the singlet-triplet splitting, the configurations of triplet multiplicity, and *B*_2*u*_ point-group symmetry, were chosen. Thus, in all the MRSDCI calculations, only the configurations consistent with the spin and point group symmetries of the targeted states are included, making the calculations strictly spin- and symmetry adapted, leading to tremendous computational savings.

In addition to the point-group and spin symmetries, CI wave functions obtained in these calculations also possess electron-hole (e–h) symmetry, which is a consequence of: (a) employing only nearest-neighbor hoppings rendering the systems bipartite, and (b) all molecules considered are half-filled, i.e., have one electron, per carbon atom. Conventionally, the 1^1^*A*_*g*_ ground state is assigned the negative (−) e-h parity, while dipole selection rules require that the one-photon excited states should possess not only opposite spatial parity (*u*, i.e., ungerade), but also opposite e-h parity, i.e., positive (+) parity. Therefore, all *B*_2*u*_/*B*_3*u*_ optically active excited states considered in this work have positive (+) e–h parity.

In smaller RGMs, all the orbitals were treated as active during the CI calculations. However, in cases of larger molecules namely RGM-50, -54, and -56, we had to resort to the frozen orbital approximation to keep the CI expansion tractable. This consists of freezing a few lowest energy occupied MOs, and removing the corresponding symmetric virtual MOs from the list, as described in our earlier works^18,19,24^.

Once CI calculations are finished, the many-body wave functions obtained are used to compute the electric dipole transition matrix elements connecting one-photon excited states to the ground state. The transition dipole elements, along with the excitation energies of the excited states, are used to compute the optical absorption cross-section *σ*(*ω*), according to the formula

3 $$\sigma (\omega )=4\pi \alpha \sum _{i}\,\frac{{\omega }_{i0}|\langle i|\hat{e}.r\mathrm{|0}\rangle {|}^{2}{\gamma }^{2}}{{({\omega }_{i0}-\omega )}^{2}+{\gamma }^{2}}.$$

In the equation above, *ω* denotes frequency of the incident light, $\hat{e}$ represents its polarization direction, ***r*** is the position operator, *α* denotes the fine structure constant, indices 0 and *i* represent, respectively, the ground and excited states, *ω*_*i*0_ is the frequency difference between those states, and *γ* is the assumed universal line width. The summation over *i*, in principle, is over an infinite number of states which are dipole connected to the ground state. However, in practice, the sum includes only those excited states whose excitation energies are within a certain cutoff, normally taken to be 8 eV.

## Results and Discussion

In order to assess the role played by electron-correlation effects on various properties of RGMs, it is important first to understand the independent particle results obtained using the tight-binding (TB) model. Therefore, in this section, we first present the result obtained from the TB model, followed by those obtained by the PPP-model.

### Tight-binding model results

**Figure 2:**
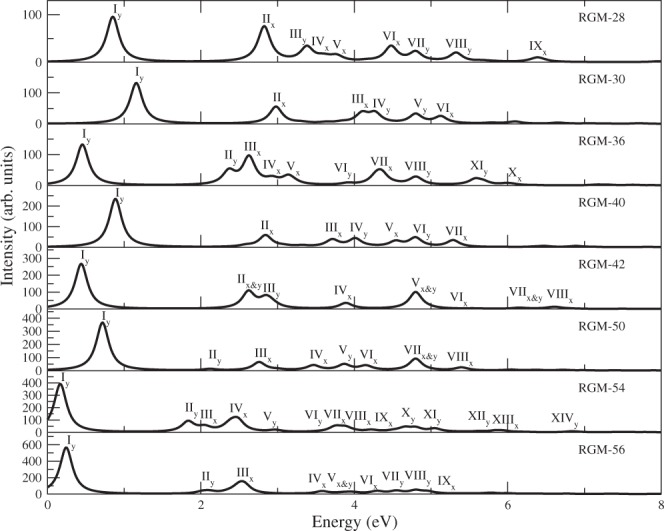
Linear optical absorption spectra for RGMs, computed using the TB model. The spectrum has been broadened with a uniform line-width of 0.1 eV.

In Fig. 2, we have presented optical absorption spectra of RGMs of varying sizes computed using the TB model. The following conclusions can be drawn from this graph:

1. The first peak in the absorption spectra for all the RGMs is *y*–polarized, and corresponds to excitation of an electron from HOMO to LUMO, leading to the excited state 1^1^*B*_2*u*_. Therefore, this peak corresponds to the optical gap of the concerned RGM, and it is the most intense peak in the spectra.
2. The intensity of the first peak in the spectra increases significantly with the increasing size of RGMs.
3. We also note that all *y*–polarized peaks are non-degenerate, and correspond to excited states of symmetry ^1^*B*_2*u*_, as per the selection rules of *D*_2*h*_ point group. All *x*–polarized peaks are doubly degenerate, as a consequence of electron-hole symmetry of the nearest neighbor TB model, and correspond to excited states of ^1^*B*_3*u*_ symmetry. For example, in RGM-30, first peak is *y*–polarized, and it is due to non-degenerate excitation $|H\to L\rangle$, while the second peak is *x*–polarized, and is due to doubly degenerate excitations $|H\to L+3\rangle$ and $|H-3\to L\rangle$. Notations *H*/*L* imply HOMO/LUMO, while *H* − *m* (*L* + *n*) imply *m*-th orbital below HOMO (*n*-th orbital above LUMO).
4. With the increasing length of the RGMs along a given orientation (zigzag or armchair), the optical gap decreases.

In Table 1, we compare the HOMO-LUMO gap obtained at the tight-binding level, with the experimental results, wherever available. For the sake of comparison, we also present the values of optical gaps obtained using the PPP-CI approach to be discussed in the next section. From the table it is obvious that the gaps obtained using the TB model are much smaller than the experimental values. On the other hand, the PPP-CI values of the optical gaps, are generally in much better agreement with the experiments. Therefore, it is obvious that the TB model cannot provide good quantitative agreement with the experiments, because it ignores the electron-correlation effects.

**Table 1:** Optical gaps of various RGMs obtained using the TB model, and the PPP model.

System	Optical gap (eV)			
	TB Model	PPP-CI		Experimental
		Scr	Std	
RGM-28	0.85	2.00	2.21	1.80^34^, 2.02^32^, 2.15^33^
RGM-30	1.16	2.11	2.43	2.14^39^, 2.21^40,41^, 2.22^35^, 2.35^36^, 2.36^37,38^
RGM-36	0.45	2.11	2.30	—
RGM-40	0.89	2.02	2.30	1.84^44^, 1.87^35,41^, 1.91^45^, 2.03^38^, 2.04^36,37^
RGM-42	0.44	1.86	2.04	—
RGM-50	0.72	1.72	1.98	1.66^35,41^
RGM-54	0.17	1.63	2.09	—
RGM-56	0.24	1.50	1.91	1.35^46^

In case of PPP model, the gaps are computed using the CI approach, by employing both the screened and the standard parameters, denoted as Scr, and Std, respectively.

### PPP model based CI results

In this section, we present our results obtained from the PPP-model based CI calculations. First, we present the results on the nature of ground state wave function of RGMs, followed by their spin gaps. Finally, we present and discuss the calculated linear optical absorption spectra of these molecules.

#### Nature of ground state

Before discussing the nature of ground state wave functions of RGMs, we would like to make a brief general comment about the number of active orbitals involved in the MRSDCI calculations performed on these molecules. For RGM-*n*, *n* = 28 to 42, all the *n* MOs of the systems were included in the MRSDCI calculations. However, from *n* = 50 to 72, it was no longer possible to perform accurate calculations with all the *n* MOs treated as active. Therefore, for these cases, some low-lying occupied MO’s, were frozen, while their unoccupied counterparts were deleted. As a result, the number of active orbitals (*N*_*act*_) for *n* = 50–56 was 42, while for *n* = 72, it was 44. The final choice of *N*_*act*_ for each molecule was decided after careful convergence considerations both for the singlet-triplet gap, and the optical absorption spectra. For *n* = 50, we have explicitly presented the convergence of optical absorption spectra with respect to *N*_*act*_.

Because, to compute the spin gaps we needed energies of 1^1^*A*_*g*_ and 1^3^*B*_2*u*_ states which are in different symmetry manifolds, we managed to perform reasonably large MRSDCI calculations, as is evident from Table S1 of Supporting Information. Therefore, we believe that the wave functions and the energies of the ground state (1^1^*A*_*g*_), and the lowest triplet state 1^3^*B*_2*u*_ are very accurate.

The dominant electronic configurations contributing to the ground state MRSDCI wave functions various RGMs are presented in Table 2. An inspection of the table reveals the following trends: (a) In all cases, the most dominant configuration to the ground state wave function is the closed-shell RHF configuration, (b) The relative magnitude of the RHF configuration to the wave function decreases significantly with the increasing sizes of RGMs. This decreases is accompanied by a significant increase in the relative contribution of the doubly-excited configuration |*H* → *L*; *H* → *L*〉 to the wave function. For example, for the smallest molecule RGM-28 the coefficients of |*HF*〉 and |*H* → *L*; *H* → *L*〉 are close to 0.80 and 0.20, respectively, while for the largest one RGM-72, they are 0.60 and 0.56, respectively, i.e., almost equal. Because these trends are true, irrespective of the Coulomb parameters used in the calculations, it is obvious that the ground states of RGMs are developing a significantly open-shell diradical character with the increasing sizes. This result was also observed in our earlier work for oligoacenes of increasing sizes, although the extent of configuration mixing for acenes was much smaller compared to RGMs^18^. Plasser *et al*.^6^ performed first-principles multi-reference averaged quadratic coupled cluster (MR-AQCC) calculations on the ground states of a large number of finite hydrogen-saturated graphene-like molecules, and computed the natural orbital (NO) occupancies of the wave functions. The molecules common between our calculations and those of Plasser *et al*.^6^ are RGM-30, RGM-42, and RGM-54. They also concluded that RGMs have a significant open-shell character. In Table S3 of the Supporting Information, we present the numbers of singly- and doubly-occupied orbitals of various irreducible representations, based upon the configurations whose coefficient are larger than 0.05 in the ground-state MRSDCI wave functions of various RGMs. These numbers are in good agreement with the number of NOs with occupancies close to one and two, reported by Plasser *et al*.^6^ for RGM-30, RGM-42, and RGM-54.

**Table 2:** Configurations making significant contributions to the ground state (1^1^*A*_*g*_) wave functions of RGM-*n* (*n* = 28–72), computed using the MRSDCI approach, and the standard (Std) and screened (Scr) parameters in the PPP-model Hamiltonian.

System	Scr	Std
RGM-28	|*HF*〉 (0.7899)	|*HF*〉 (0.8315)
	|*H* → *L*; *H* → *L*〉 (0.2264)	|*H* → *L*; *H* → *L*〉 (0.2362)
RGM-30	|*HF*〉 (0.8143)	|*HF*〉 (0.8574)
	|*H* → *L*; *H* → *L*〉 (0.1685)	|*H* → *L*; *H* → *L*〉 (0.1415)
RGM-36	|*HF*〉 (0.7269)	|*HF*〉 (0.7572)
	|*H* → *L*; *H* → *L*〉 (0.3468)	|*H* → *L*; *H* → *L*〉 (0.3399)
RGM-40	|*HF*〉 (0.7946)	|*HF*〉 (0.8401)
	|*H* → *L*; *H* → *L*〉 (0.1796)	|*H* → *L*; *H* → *L*〉 (0.1534)
RGM-42	|*HF*〉 (0.7247)	|*HF*〉 (0.7530)
	|*H* → *L*; *H* → *L*〉 (0.3508)	|*H* → *L*; *H* → *L*〉 (0.3342)
RGM-50	|*HF*〉 (0.8044)	|*HF*〉 (0.8434)
	|*H* → *L*; *H* → *L*〉 (0.2064)	|*H* → *L*; *H* → *L*〉 (0.1586)
RGM-54	|*HF*〉 (0.6504)	|*HF*〉 (0.6400)
	|*H* → *L*; *H* → *L*〉 (0.4866)	|*H* → *L*; *H* → *L*〉 (0.4975)
RGM-56	|*HF*〉 (0.6798)	|*HF*〉 (0.6724)
	|*H* → *L*; *H* → *L*〉 (0.4541)	|*H* → *L*; *H* → *L*〉 (0.4469)
RGM-72	|*HF*〉 (0.6164)	|*HF*〉 (0.5958)
	|*H* → *L*; *H* → *L*〉 (0.5562)	|*H* → *L*; *H* → *L*〉 (0.5666)

|*HF*〉 denotes the closed-shell restricted Hartree-Fock configuration, with respect to which other configurations are defined. In particular, |*H* → *L*; *H* → *L*〉 denotes the doubly-excited configuration with respect to the |*HF*〉, obtained by promoting two electrons from HOMO (*H*) to LUMO (*L*), of the concerned RGM. The expansion coefficient of each configuration in the ground state wave function is written in the parenthesis next to it.

#### Spin gaps

Spin gap of an electronic system is the energy difference between the lowest triplet and singlet states. For RGMs, the lowest singlet state is 1^1^*A*_*g*_ ground state, while the lowest triplet state is the 1^3^*B*_2*u*_ state, whose spatial part of the wave function consists predominantly of the single excitation |*H* → *L*〉, just as in the case of 1^1^*B*_2*u*_ state. Thus, at the TB level, 1^1^*B*_2*u*_ and 1^3^*B*_2*u*_ will be degenerate, and, therefore their spin and optical gaps will be identical. However, if the two gaps are found to be different for RGMs, it can only be due to electron-electron interactions. Therefore, difference in the spin and optical gaps is a measure of the electron correlation effects in RGMs. With this in mind, we computed the spin gaps of RGMs up to RGM-72, using our PPP model based MRSDCI approach. Given that the size of these CI calculations (see Table S1 of Supporting Information) is reasonably large, we believe that the spin gaps of RGMs presented in Table 3 are fairly accurate. Furthermore, in the same table, our results are compared with those reported by Horn *et al*.^31^, obtained from first-principles electron-correlated calculations performed on RGM-30, RGM-42, and RGM-54. It is obvious that the results of Horn *et al*.^31^, obtained using the *π*-electron MR-AQCC approach, are in good agreement with our results.

**Table 3:** Singlet-Triplet gaps (Δ*E*_*ST*_ = *E*(1^3^*B*_2*u*_) − *E*(1^1^*A*_*g*_)) of RGMs, computed using the MRSDCI method, employing screened (Scr) and standard parameters (Std) in the PPP model.

System	Δ*E*_*ST*_ (eV)		Δ*E*_*ST*_ (eV)
	Scr	Std	Theory (others)^31^
RGM-30	1.11	1.30	1.10^a^, 1.68^b^, 1.95^c^, 2.31^d^
RGM-40	0.97	1.16	—
RGM-50	0.79	0.94	—
RGM-28	0.76	0.75	—
RGM-42	0.40	0.36	0.30^a^, 0.26^b^, 0.23^c^, 0.21^d^
RGM-56	0.15	0.11	—
RGM-36	0.37	0.34	—
RGM-54	0.13	0.07	0.05^a^, 0.04^b^, 0.05^c^, 0.07^d^
RGM-72	0.06	0.03	—

RGMs are divided in groups of three (group one contains RGM-30, -40, -50,  group two contains RGM-28, -42, -56, and group three contains RGM -36, -54, -72), where each group corresponds to a common width, and increasing armchair length.

^a^*π*-MR-AQCC, ^b^*π*-MR-CISD + Q, ^c^*π*-MR-CISD, ^d^*π*-MCSCF.

From the inspection of Table 3 it is obvious that spin gaps of RGMs are decreasing with their increasing sizes. Thus, this result suggests that the spin gap of infinite graphene is zero, consistent with the widespread assumption that graphene is a weakly-correlated material. It is noteworthy that based on an identical PPP-MRSDCI methodology, in an earlier work from our group, it was shown that the spin gaps of oligoacenes exhibit signs of saturation with the increasing conjugation length^18^, indicating stronger electron-correlation effects. Given the fact that the oligoacenes are nothing but hydrogen-passivated, finite-sized, narrowest possible (*N*_*z*_ = 2) zigzag nanoribbons, this suggests that electron-correlation effects are stronger in them, as compared to graphene, because of their reduced dimensionality.

We present the important configurations contributing to the many body wave functions of 1^3^*B*_2*u*_ states of various RGMs in Table 4. It is obvious from the table that although the single excitation |*H* → *L*〉 makes the dominant contribution to the triplet wave function in all the cases, but other configurations also make smaller, but, significant contributions, thereby underlying the importance of including electron-correlation effects in accurate quantitative calculation of energies of triplet states.

**Table 4:** Configurations making significant contributions to the lowest triplet state (1^3^*B*_2*u*_) wave functions of RGM-*n* (*n* = 28–72), computed using the MRSDCI approach, and the standard (Std) and screened (Scr) parameters in the PPP-model Hamiltonian.

System	Scr	Std
RGM-28	|*H* → *L*〉 (0.8097)	|*H* → *L*〉 (0.8197)
	|*H* − 1 → *L* + 1〉 (0.1390)	|*H* → *L*; *H* − 2 → *L*〉 + *c*.*c*. (0.1480)
RGM-30	|*H* → *L*〉 (0.8035)	|*H* → *L*〉 (0.8049)
	|*H* − 1 → *L* + 1〉 (0.1545)	|*H* − 1 → *L* + 1〉 (0.1685)
RGM-36	|*H* → *L*〉 (0.8047)	|*H* → *L*〉 (0.8022)
	|*H* − 1 → *L* + 1〉 (0.1247)	|*H* → *L*; *H* − 2 → *L*〉 − *c*.*c*. (0.1522)
RGM-40	|*H* → *L*〉 (0.7912)	|*H* → *L*〉 (0.7876)
	|*H* − 1 → *L* + 1〉 (0.1681)	|*H* − 1 → *L* + 1〉 (0.1869)
RGM-42	|*H* → *L*〉 (0.8055)	|*H* → *L*〉 (0.7838)
	|*H* → *L*; *H* − 2 → *L*〉 + *c*.*c*. (0.1312)	|*H* → *L*; *H* − 2 → *L*〉 − *c*.*c*. (0.1922)
RGM-50	|*H* → *L*〉 (0.8060)	|*H* → *L*〉 (0.7785)
	|*H* − 1 → *L* + 1〉 (0.1224)	|*H* − 1 → *L* + 1〉 (0.1974)
RGM-54	|*H* → *L*〉 (0.8188)	|*H* → *L*〉 (0.7984)
	|*H* → *L*; *H* − 2 → *L*〉 − *c*.*c*. (0.1173)	|*H* → *L*; *H* − 2 → *L*〉 − *c*.*c*. (0.1889)
RGM-56	|*H* → *L*〉 (0.8127)	|*H* → *L*〉 (0.7697)
	|*H* → *L*; *H* − 1 → *L*〉 − *c*.*c*. (0.1496)	|*H* → *L*; *H* − 1 → *L*〉 − *c*.*c*. (0.2295)
RGM-72	|*H* → *L*〉 (0.8417)	|*H* → *L*〉 (0.8196)
	|*H* → *L*; *H* − 2 → *L*〉 + *c*.*c*. (0.1111)	|*H* → *L*; *H* − 2 → *L*〉 − *c*.*c*. (0.1738)

Various configurations are defined with respect to the closed-shell restricted Hartree-Fock configuration |*HF*〉. |*H* → *L*〉 denotes the singly-excited configuration with respect to the |*HF*〉, obtained by promoting one electron from HOMO (*H*) to LUMO (*L*), of the concerned RGM. The expansion coefficient of each configuration in the ground state wave function is written in the parenthesis next to it.

It will also be interesting to compare the experimental values of the spin-gaps of individual hydrocarbon molecules corresponding to these RGMs, with our calculated values. Therefore, we urge the experimentalists to measure the spin gaps of RGMs studied in the present work.

#### Linear optical absorption spectrum

In this section, we present optical absorption spectra of RGM-*n*, with *n* ranging from 30 to 56, computed using the PPP model, and the MRSDCI approach. Before discussing the results of our calculations, in Table S2 of Supporting Information we give the sizes of the CI matrices, for different symmetry spaces of various RGMs. The fact that the sizes of the CI matrices were in the range 2.43 × 10^5^–6.19 × 10^6^, implies that these calculations were reasonably large, and, therefore, should be fairly accurate.

**Figure 3:**
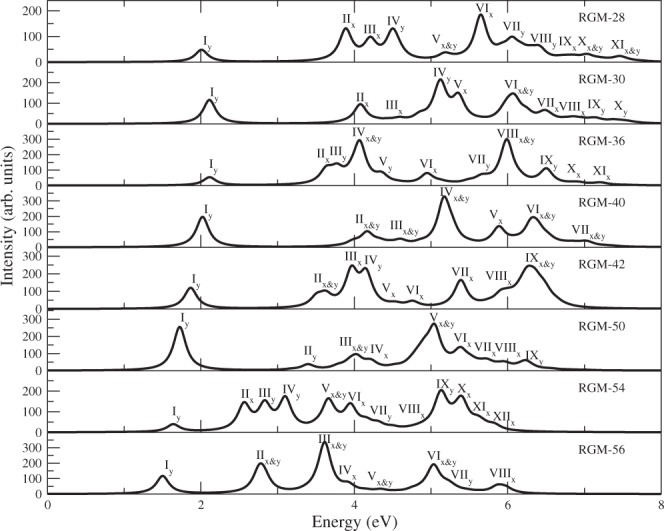
Computed linear optical absorption spectra of RGMs, obtained using the MRSDCI approach, by employing screened Coulomb parameters in the PPP model. The spectra have been broadened using a uniform line-width of 0.1 eV.

**Figure 4:**
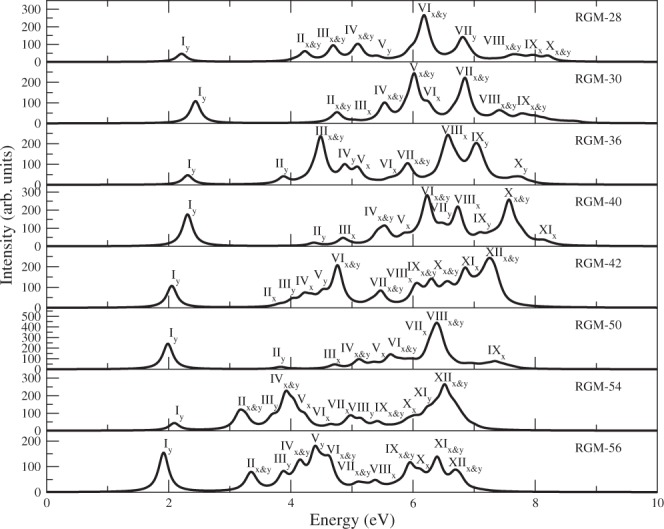
Computed linear optical absorption spectra of RGMs, obtained using the MRSDCI approach, by employing standard Coulomb parameters in the PPP model. The spectra have been broadened using a uniform line-width of 0.1 eV.

The calculated spectra of these RGMs are presented in Figs 3 and 4, while the important information regarding the excited states contributing to various peaks in the spectra, including their wave functions, are presented in Tables S12–S27 of Supporting Information.

Before discussing the spectra of individual RGMs, we discuss the general trends observed in our calculation:

1. For each RGM-*n*, the absorption spectrum obtained using the PPP-CI approach is blueshifted in comparison to the TB model.
2. For all the RGMs, absorption spectra obtained using the screened parameters are red-shifted compared to those obtained using the standard parameters.
3. In all cases, the first peak of the spectrum is due to optical excitation from the 1^1^*A*_*g*_ ground state to 1^1^*B*_2*u*_ excited state, and corresponds to the optical gap. As per electric dipole selection rules, this peak is *y*-polarized. The wave function of the 1^1^*B*_2*u*_ state for all the RGMs is dominated by singly-excited configuration |*H* → *L*〉, where *H* and *L*, respectively, denote the HOMO and the LUMO of the system.
4. For all the RGMs, the first peak is not the most intense peak. For a number of RGMs, several high energy peaks are more intense than the first one. This result is in sharp contrast with the TB model results.
5. Dominant configurations in the wave functions of the excited states corresponding to the lower energy peaks are single excitations, while those in the higher energy peaks are dominated by double and higher excitations.

In Table 5, we present the locations of the peaks corresponding to the optical gap, and a higher energy ^1^*B*_3*u*_ state with dominant contribution to the oscillator strength, obtained from our calculations. In the same table, for the sake of comparison, we also present the corresponding experimental results, and the theoretical calculations of other authors, for all the RGMs considered in this work. It is obvious from the table that the agreement between the results of our calculations, and the experimental ones, is quite good for all the RGMs. Additionally, a detailed comparison for all the important peaks of individual RGMs is presented in Tables S4–S11 of the Supporting Information. For RGM-54, we could not locate any previous experimental or theoretical data. Next, we discuss our results for the individual RGMs.

**Table 5:** Comparison of our calculations with the experiments, and the theoretical works of other authors, for the peaks corresponding to: (a) optical gap (1^1^*B*_2*u*_ state), and (b) a higher energy peak with the dominant contribution from a ^1^*B*_3*u*_ state, for various RGMs.

Excitation energy (*eV*)				
RGM	This work		Experimental	Other theoretical
	Scr. (Peak)	Std. (Peak)		
28	2.00 (*I*_*y*_) (^1^*B*_2*u*_)	2.21 (*Iy*) (^1^*B*_2*u*_)	1.80^34^, 1.98^34^, 2.02^32^, 2.15^33^, 2.43^33^,	1.47^50^ 1.78^a^^51^, 1.98^b^^51^
	4.19 (*III*_*x*_) (^1^*B*_3*u*_)	4.14 (*IIx*&*y*) (^1^*B*_2*u*_/^1^*B*_3*u*_)	4.05^34^	—
30	2.11 (*I*_*y*_) (^1^*B*_2*u*_)	2.43 (*I*_*y*_) (^1^*B*_2*u*_)	2.14^39^, 2.21^40,41^, 2.22^35^, 2.35^36^, 2.36^37,38^, 2.39^35^, 2.57^35^, 2.76^35^,	2.02^37^, 2.03^47^, 2.21^a^/2.22^c^^43^, 2.29^48^, 2.52^49^, 2.98^37^, 3.31^37^, 3.40^37^, 3.84^37^,
	5.35 (*V*_*x*_) (^1^*B*_3*u*_)	5.53 (*IVx*&*y*) (^1^*B*_2*u*_/^1^*B*_3*u*_)	5.20^37^, 5.27^36^, 5.41^37^, 5.48^35^	—
36	2.11 (*I*_*y*_) (^1^*B*_2*u*_)	2.30 (*Iy*) (^1^*B*_2*u*_)	—	—
	3.63 (*II*_*x*_) (^1^*B*_3*u*_)	3.87 (*IIy*) (^1^*B*_2*u*_)	—	3.64^42^
40	2.02 (*I*_*y*_) (^1^*B*_2*u*_)	2.30 (*I*_*y*_) (^1^*B*_2*u*_)	1.84^44^, 1.87^35,41^, 1.91^45^, 1.99^44^, 2.03^38^, 2.04^36,37^	1.65^47^, 1.67^37^, 1.79^c^/1.83^a^^43^, 1.87^45^, 1.88^48^, 2.18^49^, 2.97^37^,
	5.16 (*IV*_*x*&*y*_) (^1^*B*_2*u*_/^1^*B*_3*u*_)	5.48 (*IVx*&*y*) (^1^*B*_2*u*_/^1^*B*_3*u*_)	5.27^35^, 5.39^37^	5.30^43^
42	1.86 (*I*_*y*_) (^1^*B*_2*u*_)	2.04 (*Iy*) (^1^*B*_2*u*_)	1.41^46^, 1.57^46^	—
	3.96 (*III*_*x*_) (^1^*B*_3*u*_)	3.80 (*IIx*) (^1^*B*_3*u*_)	3.87^46^	—
50	1.72 (*I*_*y*_) (^1^*B*_2*u*_)	1.98 (*I*_*y*_) (^1^*B*_2*u*_)	1.66^35,41^	1.40^47^, 1.51^c^/1.54^*a*^^43^, 1.60^48^, 1.97^49^
	4.97 (*V*_*x*&*y*_) (^1^*B*_2*u*_/^1^*B*_3*u*_)	5.12 (*IVx*&*y*) (^1^*B*_2*u*_/^1^*B*_3*u*_)	4.80^35^	5.2^43^
54	1.63 (*I*_*y*_) (^1^*B*_2*u*_)	2.09 (*Iy*) (^1^*B*_2*u*_)	—	—
	2.56 (*II*_*x*_) (^1^*B*_3*u*_)	3.20 (*IIx*&*y*) (^1^*B*_2*u*_/^1^*B*_3*u*_)	—	—
56	1.50 (*I*_*y*_) (^1^*B*_2*u*_)	1.91 (*Iy*) (^1^*B*_2*u*_)	1.35^46^, 2.01^46^, 2.10^46^,	—
	2.79 (*II*_*x*&*y*_) (^1^*B*_2*u*_/^1^*B*_3*u*_)	3.35 (*IIx*&*y*) (^1^*B*_2*u*_/^1^*B*_3*u*_)	3.21^46^	—

Our calculations were performed using the PPP-MRSDCI approach, employing both the screened (Scr.) and the standard (Std.) parameters. All results are in eV units. ^a^TDDFT method, ^b^TDPPP method, ^c^DFT(Kohan-Sham) method.

#### RGM-28

Clar and Schmidt^32^, Arabei *et al*.^33^, and Konishi *et al*.^34^ have reported the measurements of the absorption spectrum of bisanthene, and its derivatives, the structural analogs of RGM-28. In Figs 3 and 4, we present our calculated spectra using the screened and standard parameters, respectively, within the PPP-CI approach. If we compare the relative intensity of the first peak of the experimental spectra, we find that results of Arabei *et al*.^33^ are in perfect agreement with our results in that the first peak is not the most intense. However, Konishi *et al*.^34^ report that the first peak is the most intense one, in complete disagreement with our results. Our calculated location of the first peak corresponding to the optical gap, was found to be 2.00 eV with the screened parameters, and 2.21 eV for the standard parameters. As is obvious from Table 5, the experimental values of the optical gap range from 1.80 eV to 2.15 eV. Thus, we find that both our screened and standard parameter of optical gap are quite close to the range of experimental values. We also note that our screened parameter of 2.00 eV is in almost perfect agreement with the value of optical gap 2.02 eV, measured by Clar and Schmidt^32^. As far as higher energy peaks are concerned, Konishi *et al*.^34^ report a peak at 4.05 eV, which is in good agreement with our standard parameter peak computed at 4.14 eV, while the corresponding screened parameter candidate at 4.19 eV is somewhat higher. Our calculation predicts several more peaks, whose details are given in Table S4 of Supporting Information. We note that some of these peaks are in an energy range, for which no experimental results exist. We hope that in future measurements of the absorption spectrum of bisanthenes, energy range of 5 eV, and beyond, will be explored.

Peak VI is the most intense peak in the absorption spectra computed using both the screened as well as the standard parameters. The most intense peak computed using the screened parameters located at 5.63 eV, corresponds to a state with *B*_3*u*_ symmetry, whose wave function is dominated by $|H-2\to L+4\rangle -cc.$ excitations, where cc. denotes the charge conjugated configration. However, the standard parameter calculations predict the most intense intense peak to be due to a *B*_3*u*_ state, located at 5.95 eV, along with a small mixture of a *B*_2*u*_ state located at 6.17 eV, with their wave functions dominated by single excitations $|H-2\to L+4\rangle -cc.$, and $|H-3\to L+3\rangle$, respectively. The detailed wave function analysis of all the excited states contributing to various peaks in the calculated spectra of RGM-28, is presented in Tables S12 and S13 of the Supporting Information.

#### RGM-30

Koch *et al*.^35^, Ruiterkamp *et al*.^36^ and Halasinski *et al*.^37^ have reported the measurements of the absorption spectrum of terrylene, the structural analog of RGM-30, and its derivatives. However, Clar *et al*.^38^, Kummer *et al*.^39^, Biktchantaev *et al*.^40^ and Baumgarten *et al*.^41^ reported only the optical gap of terrylene. In Figs 3 and 4, we present our calculated spectra using the screened and standard parameters, respectively, within the PPP-CI approach. If we compare the relative intensity of the first peak of the experimental spectra, we find that the results of Koch *et al*.^35^, Ruiterkamp *et al*.^36^ and Halasinski *et al*.^37^ are in perfect agreement with our results in that the first peak is not the most intense. The calculated location of the first peak of the absorption spectrum, which defines the optical gap, was found to be 2.11 eV, and 2.43 eV, from our standard, and screened parameter based calculations, respectively. As it is obvious from Table 5, that the experimental values of the optical gap range from 2.14 eV to 2.36 eV. Thus, we find that both our screened and standard parameter of optical gap are quite close to the range of experimental values. We also note that our screened parameter value of 2.11 eV is in almost perfect agreement with the value of optical gap 2.14 eV, measured by Kummer *et al*.^39^. As far as higher energy peaks are concerned, Halasinski *et al*.^37^ report a peak at 5.41 eV, in good agreement with our screened parameter peak computed at 5.35 eV, while the corresponding standard parameter candidate at 5.53 eV is in good agreement with a peak at 5.48 eV, measured by Koch *et al*.^35^. Our calculation predicts several more peaks, whose details are given in Table S5 of Supporting Information. Furthermore, we have computed several peaks located beyond 7 eV, for which no experimental results exist. We hope that in future measurements of the absorption spectrum of terrylene, this higher energy range will be explored.

In the spectra computed using the screened parameters, IV peak is the most intense, and it is due to a *B*_2*u*_ state, located at 5.12 eV, whose wave function is dominated by the $|H-2\to L+2\rangle$ excitation. For the standard parameter calculations, peak V is the most intense one, due to a *B*_2*u*_ state located at 6.01 eV, along with a small mixture of *B*_3*u*_ state located at 5.87 eV, with wave functions dominated by configurations $|H-3\to L+3\rangle$, and $|H\to L;H\to L+4\rangle -cc.$, respectively. The detailed wave analysis of all the excited states contributing peaks in the computed spectra is presented in Tables S14 and S15 of the Supporting Information.

#### RGM-36

The hydrogen passivated structural analog of RGM-36 is tetrabenzocoronene, for which we were unable to locate any experimentally measured optical absorption spectrum. Therefore, we can only compare our calculations to the theoretical works of other authors, for which also we could find just one TDDFT based computation of the optical absorption spectra by Malloci *et al*.^42^. In Figs 3 and 4, we present our calculated spectra using the screened and standard parameters, respectively, within the PPP-CI approach. If we compare the relative intensity of the first peak in the spectra, we find that the results of Malloci *et al*.^42^ are in perfect agreement with our results in that the first peak is not the most intense. In Table 5, we have compared the locations of various peaks reported by Malloci *et al*. with our computed results. We find that the value of optical gap reported by Malloci *et al*.^43^ is 0.95 eV, which is significantly smaller than our computed results of 2.11 eV (screened) and 2.30 eV (standard). Given such severe disagreement between two theoretical calculations, it will be really useful if an experiment is performed on this molecule, or another theoretical calculation is done. Given the fact that our results on optical gaps on smaller RGMs were in excellent agreement with the experiments, we speculate that the TDDFT calculation of Malloci *et al*.^42^ has significantly underestimated the optical gap of RGM-36. As far as higher peaks are concerned, our screened parameter calculations predict a peak at 3.63 eV due to a ^1^*B*_3*u*_ state, whose location is in perfect agreement with a peak at 3.64 eV, reported by Malloci *et al*.^42^. Our calculation predicts several more peaks, whose details are given in Table S6 of Supporting Information. As far as higher energy peaks computed by Malloci *et al*.^42^ are concerned, our PPP model values are generally in good agreement with them.

For both the screened as well as the standard parameters computed absorption spectra, peak VIII is the most intense one. In the spectra computed using the screened parameters, the most intense peak is located at 5.99 eV, and is due to states of symmetries *B*_2*u*_ and *B*_3*u*_ contributing almost equally to the oscillator strength, with their many-particle wave functions dominated by configurations $|H-3\to L+3\rangle$, and $|H-2\to L+5\rangle +cc.$, respectively. In the spectrum computed using the standard parameters, the most intense peak is located at 6.57 eV, due to a *B*_3*u*_ state, with wave function dominated by $|H-5\to L+2\rangle +cc.$ excitations. The detailed wave analysis of excited states contributing peaks in the spectra computed by the screened, and the standard parameters is presented in Tables S16 and S17 of the Supporting Information.

#### RGM-40

Ruiterkamp *et al*.^36^, Koch *et al*.^35^, and Halasinski *et al*.^37^ have reported the measurements of the absorption spectrum of quaterrylene, the hydrogen passivated structural analog of RGM-40, and its derivatives. However, Clar *et al*.^38^, Former *et al*.^44^, Gudipati *et al*.^45^, and Baumgarten *et al*.^41^ reported only the optical gap of quaterrylene. In Figs 3 and 4, we present our calculated spectra using the screened and the standard parameters, respectively, within the PPP-CI approach. If we compare the relative intensity of the first peak of the experimental spectra, we find that results of Ruiterkamp *et al*.^36^ and Halasinski *et al*.^37^ are in perfect agreement with our results in that the first peak is not the most intense. However, Koch *et al*.^35^ report that the first peak is the most intense one, in disagreement with our results, as well those of other experimentalists. As is obvious from Table 5, the experimental values of the optical gap range from 1.87 eV to 2.04 eV, implying that our screened parameter result of optical gap (2.02 eV) is quite close to the range of experimental values, while the optical gap obtained using the standard parameters (2.30 eV) is somewhat larger. We also note that our screened parameter value of the optical gap, 2.02 eV, is in almost perfect agreement with 2.03 eV, the value of the optical gap measured by Clar and Schmidt^32^. As far as the higher energy peaks are concerned, Koch *et al*.^35^ report a peak at 5.27 eV, in good agreement with our screened-parameter based peak at 5.16 eV. Halasinski *et al*.^37^ report a peak at 5.39 eV, which is in good agreement with the peak at 5.48 eV, predicted by standard parameter calculations. Our calculation predicts several more peaks, whose details are given in Table S7 of Supporting Information. Furthermore, Halasinski *et al*.^37^ have measured four more peaks in the range 5.82–6.63 eV, each of which is in good agreement with our calculated peaks (see Table S7 of Supporting Information).

In the spectra computed using the screened parameters, peak IV is most intense, and it is due to a state of *B*_2*u*_ symmetry, located at 5.16 eV, along with a small contribution from a *B*_3*u*_ state, located at 5.17 eV. The wave functions of these states are dominated by single excitations $|H-2\to L+2\rangle$, and $|H-1\to L+5\rangle -cc.$, respectively. For the standard parameter calculations, peak VI is the most intense one, corresponding again to a mixture of a *B*_2*u*_ state (at 6.23 eV), and a *B*_3*u*_ state (at 6.17 eV), with wave functions dominated by excitations $|H-4\to L+4\rangle ,$ and $|H\to L+\mathrm{1;}\,H-6\to L\rangle -cc.$, respectively. The detailed wave function analysis of the excited states contributing to various peaks in the spectra calculated by the screened and standard parameters, respectively, is presented in Tables S18 and S19 of the Supporting Information.

#### RGM-42

Teranthene is the hydrogen-saturated structural analogue of RGM-42, for which no experimental, or theoretical data is available, as far as optical absorption spectrum is concerned. However, Konishi *et al*.^46^ have reported the measurement of the absorption spectrum of teranthene with tertiary-butyl group attached on its edge atoms, and the results of their experiments, along with those obtained from our calculations, are summarized in Table 5. In Figs 3 and 4, we present our calculated spectra of RGM-42 using the screened, and the standard parameters, respectively, within the PPP-CI approach. If we compare the relative intensity of the first peak of the experimental spectra, we find that results of Konishi *et al*.^46^ are in perfect agreement with our results in that the first peak is not the most intense. However, as is obvious from Table 5, quantitatively speaking, our theoretical results and experimental results of Konishi *et al*.^46^ disagree completely in the low-energy region. Konishi *et al*.^46^. have reported two low-lying excited energy peaks located at 1.17 eV, and 1.21 eV, for which there are no counterparts in our computed spectra. The calculated location of the first peak, which also corresponds to the optical gap, was found to be 1.86 eV from our screened parameter calculation, and 2.04 eV for the standard parameter calculation, as against significantly smaller values 1.17–1.21 eV measured by Konishi *et al*.^46^. As far as higher energy peaks are concerned, Konishi *et al*.^46^ report a peak at 3.87 eV, which is in good agreement both with a screened parameter peak at 3.96 eV, and a standard parameter peak at 3.80 eV. Our calculations predict several peaks in the energy region of 4 eV and beyond (see Table S8 of Supporting Information for details), which Konishi *et al*.^46^ have not probed. The only possible reason we can think of behind the disagreement between the theory and the experiments in the lower energy region is that the experiments were performed on teranthene saturated with t-butyl group, while our results are valid for hydrogen-saturated material. Nevertheless, we hope that more groups will perform measurements of optical absorption spectra of teranthenes so as to be sure about the value of their optical gap.

In the spectrum computed using the screened parameters, peak III is most intense, corresponding to a *B*_3*u*_ state located at 3.96, whose wave function is dominated by $|H-3\to L\rangle -\,cc.$ single excitations. For the standard parameter calculation, peak VII is most intense, corresponding again to a *B*_3*u*_ state, but located at 5.47 eV, with a small mixture of *B*_2*u*_ state located at 5.33 eV. The wave functions of the two states are dominated by single excitations, $|H-3\to L\rangle -cc.$, and $|H-1\to L+1\rangle$, respectively. The detailed wave analysis of all the excited states contributing to peaks in the calculated spectra using the screened and the standard paremeters, is presented in Tables S20 and S21, respectively, of the Supporting Information.

#### RGM-50

**Figure 5:**
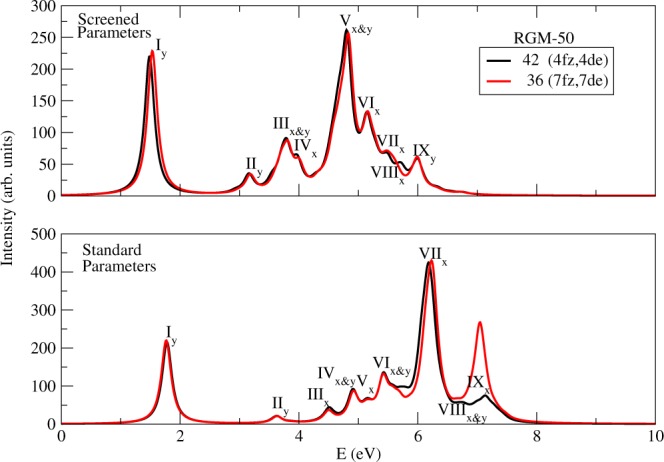
Convergence of the calculated spectra of RGM-50, with respect to the number of active orbitals in the MRSDCI calculations. Curve in black color corresponds to the calculation with 42 active orbitals (4 frozen and 4 deleted orbitals), while that in red is from a calculation with 36 active orbitals (7 frozen and 7 deleted orbitals).

For RGM-50 and larger structures, it would have been computationally very tedious to perform MRSDCI calculations retaining all the MOs, therefore, we decided to freeze a few lowest-lying occupied orbitals, and delete their electron-hole symmetric highest virtual orbitals. For the case of RGM-50, we froze/deleted four occupied/virtual orbitals, so that the total number of MOs involved in the calculations reduced to forty-two, same as in case of RGM-42. In order to demonstrate that this act of freezing and deleting the MOs does not affect the calculated optical absorption spectra, we have performed the calculations for RGM-50, with four and seven frozen/deleted orbitals, leading to 42/36 active MOs. From the calculated absorption spectra presented in Fig. 5, it is obvious that except for the intensity of the highest energy peak IX in the standard parameter calculations, the spectra remain the same for both the cases, implying that the convergence has been achieved within an acceptable tolerance.

Hydrogen saturated structural analog of RGM-50 is pentarylene, for which, to the best of our knowledge, no experimental data of optical absorption exists. However, Koch *et al*.^35^ have reported the measurements of the absorption spectrum of pentarylene saturated with the t-butyl group, while Baumgarten *et al*.^41^ measured only its optical gap. We present our calculated absorption spectra for RGM-50 in Figs 3 and 4, while the comparison of important peak locations resulting from our calculations, with the experiments, and other theoretical works is presented in Table 5. The value of the optical gap measured by both the groups^35,41^ is 1.66 eV, which is in very good agreement with the value 1.72 eV computed using the screened parameters, while the corresponding standard parameter value 1.98 eV is on the higher side. If we compare the relative intensity of the first peak of the experimental spectra corresponding to the optical gap, we find that Koch *et al*.^35^ report that the first peak to be the most intense one, in disagreement with our results. However, the noteworthy point is that the first peak computed using the screened parameter, is quite intense, and is only somewhat lesser in intensity than the most intense peak (peak V) of the computed spectrum. As far as higher energy peaks are concerned, Koch *et al*.^35^ report a peak at 4.80 eV, which is somewhat close to our screened parameter peak located at 4.97 eV. Furthermore, we have computed several higher energy peaks as well, for which no experimental results exist. We hope that in future measurements of the absorption spectrum of pentarylene, energy range beyond 5.3 eV will be explored.

On comparing our results to the calculations by other authors, we find that the value of the optical gaps reported by Viruela-Martín *et al*.^47^ (1.40 eV) using the valence-effective Hamiltonian approach, Malloci *et al*.^43^ (1.54 eV) using the TDDFT method are significantly smaller than our results, as well as experiments. However, Minami *et al*.^48^ report a TDDFT value which is in good agreement with the experiment value of the optical gap, but about 0.1 eV lower than our result. Karabunarliev *et al*.^49^ computed the optical gap to be 1.97 eV using PM3 semi-empirical method, is in perfect agreement with our standard parameter result located at 1.98 eV, but significantly higher than the experimental value, as well as our screened parameter value. As far as higher energy peaks computed by Malloci *et al*. are concerned, our PPP model values are in reasonable agreement with them.

In the spectra computed using the screened parameter, peak V is most intense, and is due to a *B*_2*u*_ state located at 5.04 eV, along with a small intensity due to a *B*_3*u*_ state located at 4.90 eV. The wave functions of the two states are dominated by configurations $|H-3\to L+3\rangle$, and $|H-5\to L+1\rangle +cc.$ excitations, respectively. In the standard parameter spectrum, peak VIII is most intense, and is mainly due to a *B*_2*u*_ state located at 6.38 eV, along with a small contribution of a *B*_3*u*_ state located at 6.45 eV. The dominant contributions to the many-particle wave functions of these two states are from single excitation $|H-4\to L+4\rangle$, and the double excitation $|H\to L+\mathrm{6;}\,H\to L+1\rangle -cc.$, respectively. The detailed wave function analysis of all the excited states contributing peaks in the spectra computed using the screened and the standard parameters, are presented in Tables S22 and S23, respectively, the Supporting Information.

#### RGM-54

For the case of RGM-54, we performed MRSDCI calculations after freezing/deleting six occupied/virtual MOs, i.e., with forty two active MOs. For this molecule, we were not able to locate any experimental results, or other theoretical calculations, thus, making our results the first ones. We hope that our calculations will give rise to future theoretical and experimental works on this system.

Calculated optical absorption spectra for RGM-54 are presented in Figs 3 and 4, obtained using the screened and standard parameters, respectively. The locations of important peaks, and the symmetries of excited states giving rise to them, are summarized in Table S10 of Supporting Information. The first peak corresponding to the optical gap (see Table 5), is a very weak peak from both sets of calculations, and was found to be at 1.63 eV with the screened parameters, and 2.09 eV with the standard parameters. Given the pattern observed for smaller RGMs discussed in the previous sections, we expect the screened parameter value of the optical gap to be closer to the experimental value.

In the screened parameter calculations, next we find a group of three well-separated peaks, with strong, and almost equal, intensities, located at 2.56 eV, 2.83 eV, and 3.09 eV. The first of these peaks corresponds to an *x*-polarized transition, while the next two are *y*-polarized. In the standard parameter spectrum as well, the next three peaks are quite strong, and well separated, but they have their intensities in the ascending order, while the middle peak (peak III) appears as a shoulder of peak IV. The locations of these peaks are blue-shifted compared to their screened parameter counterparts, and are 3.20 eV, 3.69 eV, and 3.98 eV. The polarization characteristics are also different, with two of the peaks exhibiting mixed polarization.

At higher energies, in the screened parameter spectrum there are well defined high-intensity peaks at energies 3.71 eV (*x*/*y* polarized), 3.95 eV (*x* polarized), 5.14 eV (*y* polarized), and 5.40 eV (*x* polarized). Out of these the peak located at 5.14 eV (peak IX) is the most intense peak of the computed spectrum. This peak is due to a *B*_2*u*_ state, whose wave function is dominated by the $|H-3\to L+3\rangle$ singly-excited configuration.

In the standard parameter spectrum, beyond 4 eV, there are a number of low-intensity peaks or shoulders, except for a peak located at 6.56 eV (peak XII), which is the most intense one, and exhibits mixed polarization. This peak is due to a *B*_3*u*_ state located at 6.51 eV, along with a smaller contribution to the intensity from a *B*_2*u*_ state located at 6.61 eV. The wave functions of the two states are dominated by singly excited configurations, $|H-2\to L+7\rangle +cc.$, and $|H-3\to L+3\rangle$, respectively.

The detailed wave function analysis of all the excited states contributing peaks in the spectra computed using the standard parameters, and the screened parameters, are presented in Tables S24 and S25, respectively, of the Supporting Information.

#### RGM-56

Again, due to a large number of electrons in the system, for RGM-56 we froze/deleted seven occupied/virtual orbitals, so that the total number of active MOs involved in the calculations reduced to forty-two, same as in case of RGM-42, RGM-50, and RGM-54.

The hydrogen saturated analog of RGM-56 is quateranthene, for which no experimental measurements of optical absorption spectrum exist. However, Konishi *et al*.^46^ measured the absorption spectrum of quateranthene, with t-butyl groups attached to its edge carbon atoms, with which we will compare our calculated spectra. In Figs 2 and 3, we present our calculated spectra using the screened and standard parameters, respectively, within the PPP-MRSDCI approach. In Tables 5, we present the locations of various peaks in the calculated spectra, and compare them to the measured values of Konishi *et al*.^46^. If we compare the relative intensity of the first peak of the experimental spectra, we find that the results of Konishi *et al*.^46^ are in perfect agreement with ours in that the first peak is not the most intense. The calculated location of the first peak, which also corresponds to the optical gap, from our calculations was found to be 1.50 eV with the screened parameters, and 1.91 eV with the standard parameters. The experimental value of optical gap reported by Konishi *et al*.^46^ is 1.35 eV, which is about 0.15 eV lower than our screened parameter value, but significantly smaller than the value obtained from the standard parameter calculations. As far as higher energy peaks are concerned, Konishi *et al*.^46^ report a peak at 3.21 eV, in good agreement with our standard parameter peak computed at 3.35 eV. Our calculation predicts several more peaks, whose details are given in Table S11 of Supporting Information. We hope that in future measurements of the absorption spectrum of quateranthene, energy range beyond 3.50 eV will be explored.

In the spectra computed using screened parameter, peak III is the most intense one, and it is due to a *B*_2*u*_ state located at 2.76 eV, with a smaller contribution to the intensity from a *B*_3*u*_ state located at 2.82 eV. The wave functions of the two states are dominated by doubly-excited configurations $|H\to L;H\to L+1\rangle -cc.$, and $|H\to L;H-2\to L\rangle -cc.$, respectively. In the standard parameter spectrum, peak V is most intense, and is entirely due to a *B*_2*u*_ state located at 4.34 eV, whose wave function derives most important contribution from the singly-excited configuration $|H-2\to L+2\rangle$. The detailed wave function analysis of the excited states contributing to varioius peaks in the spectra computed using the screened and standard parameters is presented in Tables S26 and S27, respectively, of the Supporting Information.

## Conclusions

In this paper, we have presented the results of our correlated-electron calculations of spin gaps and optical absorption spectra of rectangular graphene-like polycyclic aromatic molecules, with the number of carbon atoms in the range 28–56, using PPP model Hamiltonian, and the MRSDCI approach. We analyzed the ground state wave functions of these molecules, and found that with the increasing size, they exhibit significant configuration mixing leading to diradical open-shell character. Results of our calculations on the spin gaps of these RGMs, when extrapolated to infinite graphene, suggest that it has a vanishing spin gap, implying weak electron correlation effects. This result is consistent with the widespread assumption that graphene is a weakly-correlated material.

For the case of optical absorption spectra, we generally found very good agreement with the experiments performed on hydrogen-saturated structural analog of each RGM, wherever experimental data was available. In certain cases, where no experimental data was available for the H-passivated molecule, the comparison was instead made with the measurements performed on t-butyl group saturated systems, and some quantitative disagreements were encountered, most severe of which were for RGM-42. It will be very interesting if future experimental measurements could be performed on the H-passivated molecules in those cases. For the case of RGM-36, and RGM-54, no experimental measurements exist, while for the case of RGM-54, even prior theoretical calculations do not exist. Thus, results of our calculations on these molecules could be tested in future measurements of their absorption spectra.

## Supplementary information

Supporting Information: Excited States and Optical Properties of Hydrogen-Passivated Rectangular Graphenes: A Computational Study
