# Supplementary material for: Excited States and Optical Properties of Hydrogen-Passivated Rectangular Graphenes: A Computational Study
Source: Sci Rep. 2019 May 28;9:7958. doi: 10.1038/s41598-019-44258-4 (PMC6538642; doi:10.1038/s41598-019-44258-4)
Supplement: Supplementary file 1 — Supporting Information: Excited States and Optical Properties of Hydrogen-Passivated Rectangular Graphenes: A Computational Study [file 41598_2019_44258_MOESM1_ESM.pdf]

Supporting Information

Excited States and Optical Properties of Hydrogen-Passivated Rectangular Graphenes: A Computational Study

Deepak Kumar Rai[1] **and Alok Shukla**[1,*]

[1]Department of Physics, Indian Institute of Technology Bombay, Powai, Mumbai 400076, India
[*]shukla@phy.iitb.ac.in

ABSTRACT

This file contains important information related to various aspects of our calculations.

Size of the CI matrices

In order to explain the large-scale nature of these calculations, in Tables S1 and S2 and we present the dimensions of the CI matrices employed in these calculations, for various symmetry subspaces, of different RGMs. The CI calculations are performed using MRSDCI method, by employing PPP Coulomb parameters, and the point group symmetry of the concerned RGMs, are also indicated in the table.

Table S1. Dimensions of the CI matrices (N_{total}) for different symmetry subspaces, employed in the calculations of spin gaps of RGMs containing n carbon atoms.

n	1^1A_g	1^3B_{2u}
	N_{total}	N_{total}
28	1630819^a	3363548^a
	929383^b	2052250^b
30	1271636^a	2516476^a
	1355882^b	2224033^b
36	2895688^a	6999314^a
	3470683^b	8213570^b
40	5346813^a	9103631^a
	3897268^b	4429759^b
42	4690547^a	8964411^a
	5694171^b	10937840^b
50	3504598^a	3371187^a
	2655705^b	5103671^b
54	4479514^a	10745638^a
	3949223^b	9923969^b
56	3913511^a	10766994^a
	4181480^b	9476458^b
72	4180503^a	10475673^a
	4171695^b	9450095^b

MRSDCI^a method with screened parameters.
MRSDCI^b method with standard parameters.

Table S2. Dimension of the CI matrices (N_{total}) of different symmetry subspaces involved in the MRSDCI calculations of the optical absorption spectra of RGMs, containing n carbon atoms.

n	^1A_g	$^1B_{2u}$	$^1B_{3u}$
	N_{total}	N_{total}	N_{total}
28	243473^a	1286950^a	1195300^a
	176670^b	1304940^b	1363812^b
30	148576^a	1417769^a	1128568^a
	210078^b	1650565^b	1220974^b
36	1093085^a	1309198^a	1506432^a
	1149578^b	1983952^b	1607780^b
40	2675017^a	4768452^a	3136672^a
	3897268^b	6200883^b	4820202^b
42	2729332^a	3551497^a	3195172^a
	3477479^b	395229^b	4042750^b
50	3504598^a	4494916^a	5083760^a
	2655705^b	5637511^b	6193064^b
54	2150043^a	2473193^a	3038570^a
	3047218^b	4003216^b	357911^b
56	2125583^a	2983416^a	3013058^a
	2830899^b	3086912^b	3308186^b

MRSDCI^a method with screened parameters.
MRSDCI^b method with standard parameters.

Orbital Occupation

Table S3. The table presents the number of reference configurations (N_{ref}) used for the MRSDCI calculations for the ground state of various RGMs, performed using both the standard and the screened parameters in the PPP model. Additionally, the total number of doubly and singly occupied orbitals in the reference configurations, classified according to their irreducible representations (irreps) of the D_{2h} point group, are also presented for each calculation. Those configurations are chosen as reference configurations, if the magnitude of their coefficient in the ground state wave functions is at least 0.05.

System	Screened parameters						Standard parameters					
	N_{ref}	Orbital Occupancy	Orbital Irreps				N_{ref}	Orbital Occupancy	Orbital Irreps			
			^1A_g	$^1B_{3u}$	$^1B_{2u}$	$^1B_{1g}$			^1A_g	$^1B_{3u}$	$^1B_{2u}$	$^1B_{1g}$
RGM-28	12	Double	3	2	2	2	9	Double	3	2	2	2
		Single	1	1	2	1		Single	1	1	2	1
RGM-30	6	Double	4	2	3	1	8	Double	4	2	3	1
		Single	1	1	1	2		Single	1	1	1	2
RGM-36	4	Double	5	3	4	3	6	Double	4	3	4	2
		Single	0	1	1	1		Single	1	1	1	2
RGM-40	5	Double	6	3	5	2	7	Double	5	3	5	2
		Single	0	1	1	2		Single	1	1	1	2
RGM-42	4	Double	5	5	5	3	6	Double	5	4	4	3
		Single	1	0	1	1		Single	1	1	2	1
RGM-50	4	Double	8	4	7	3	4	Double	8	4	7	3
		Single	0	1	0	2		Single	0	1	0	2
RGM-54	5	Double	7	5	6	5	7	Double	7	5	6	5
		Single	1	1	1	1		Single	1	1	1	1
RGM-56	5	Double	7	6	6	5	7	Double	7	6	6	5
		Single	1	0	2	1		Single	1	0	2	1
RGM-72	8	Double	9	7	9	7	8	Double	9	7	9	6
		Single	1	1	1	1		Single	1	1	1	2

Comparison of Computed peaks Locations for RGMs with the Experiments and Other Theoretical Calculations

Table S4. Comparison of calculated peak locations in the optical absorption spectra of RGM-28, with the experimental, and other theoretical results, for bisanthene. The calculations were performed using the PPP-MRSDCI approach, employing both the screened and the standard parameters. All results are in eV units.

Experiments	Theory (others)	This work	
		Screened	Standard
1.80[1],1.98[1],2.02[2], 2.15[4],2.43[4],	1.47[3] 1.78[a5], 1.98[b5]	2.00 ($^1B_{2u}$)	2.21($^1B_{2u}$)
-	2.83[a5], 2.96[b5]	-	-
3.64[1],3.87[4]	3.76[3]	3.87 ($^1B_{3u}$)	-
4.05[1]	-	4.19($^1B_{3u}$)	4.14($^1B_{2u}/^1B_{3u}$)
4.80[4]	4.47[3]	4.49($^1B_{2u}$)	4.68 ($^1B_{2u}/^1B_{3u}$)
-	5.39[3]	5.22($^1B_{2u}/^1B_{3u}$)	5.09 ($^1B_{2u}/^1B_{3u}$)
-	-	5.63($^1B_{3u}$)	5.41($^1B_{2u}$)
-	-	6.05($^1B_{2u}$)	6.06 ($^1B_{2u}/^1B_{3u}$)
-	6.35[3]	6.41($^1B_{2u}$)	-
-	7.09[3]	7.03($^1B_{2u}/^1B_{3u}$)	6.80($^1B_{2u}$)
[a] TDDFT method, [b] TDPPP method			

Table S5. Comparison of calculated peak locations in the optical absorption spectra of RGM-30, with the experimental, and other theoretical results, for terrylene. The calculations were performed using the PPP-MRSDCI approach, employing both the screened and the standard parameters. All results are in eV units.

Experiments	Theory (others)	This work	
		Screened	Standard
2.14[6], 2.21[7,8], 2.22[11], 2.35[12], 2.36[9,15], 2.39[11], 2.57[11], 2.76[11], -	2.02[9], 2.03[10], 2.21[a]/2.22[c13], 2.29[14], 2.52[16], 2.98[9], 3.31[9], 3.40[9], 3.84[9],	2.11 ($^1B_{2u}$)	2.43 ($^1B_{2u}$)
-	-	4.07 ($^1B_{3u}$)	-
4.33[9], 4.47[11]	-	4.58 ($^1B_{3u}$)	4.64 ($^1B_{2u}$)
4.71[9,12]	4.7[13]	-	4.75($^1B_{3u}$)
-	-	5.12 ($^1B_{2u}$)	5.03($^1B_{3u}$)
5.20[9], 5.27[12], 5.41[9], 5.48[11]	- -	5.35 ($^1B_{3u}$)	5.53 ($^1B_{2u}/^1B_{3u}$)
-	-	5.95 ($^1B_{2u}$)	5.87 ($^1B_{3u}$)
6.10[11], 6.19[9],	6[13]	6.08 ($^1B_{3u}$)	6.01 ($^1B_{2u}$)
6.44[9],	-	6.47 ($^1B_{3u}$)	6.24 ($^1B_{3u}$)
6.69[9],	6.8[13]	6.86 ($^1B_{3u}$)	6.76 ($^1B_{3u}$)
[a]TDDFT method, [c]DFT(Kohan-Sham) method			

Table S6. Comparison of calculated peak locations in the optical absorption spectra of RGM-36, with theoretical results of other authors for tetrabenzocoronene. No experimental results are available for this molecule. The calculations were performed using the PPP-MRSDCI approach, employing both the screened and the standard parameters. All results are in eV units.

Theory (others)[17]	This work	
	Screened	Standard
0.95	-	-
-	2.11 ($^1B_{2u}$)	2.30($^1B_{2u}$)
3.16	-	-
3.64	3.63($^1B_{3u}$)	-
-	3.77($^1B_{2u}$)	3.87($^1B_{2u}$)
-	4.01($^1B_{2u}/^1B_{3u}$)	-
-	4.35($^1B_{2u}$)	4.41($^1B_{2u}/^1B_{3u}$)
4.83	4.94($^1B_{3u}$)	4.88($^1B_{2u}$)
-	-	5.09($^1B_{3u}$)
-	5.65($^1B_{2u}$)	5.60($^1B_{3u}$)
-	5.99($^1B_{2u}/^1B_{3u}$)	5.86($^1B_{2u}/^1B_{3u}$)
6.22	6.50($^1B_{2u}$)	6.57($^1B_{2u}$)

Table S7. Comparison of calculated peak locations in the optical absorption spectra of RGM-40, with the experimental, and other theoretical results, for quaterrylene. The calculations were performed using the PPP-MRSDCI approach, employing both the screened and the standard parameters. All results are in eV units.

Experiment	Theory (others)	This work	
		Screened	Standard
1.84[18], 1.87[7,11], 1.91[19], 1.99[18], 2.03[15], 2.04[9,12],	1.65[10],1.67[9], 1.79[c]/1.83[a][13], 1.87[19], 1.88[14], 2.18[16], 2.97[9], 3.13[9], 3.25[9], 3.40[9],	2.02 ($^1B_{2u}$)	2.30 ($^1B_{2u}$)
3.71[11], 3.78[9], 3.85[11], 3.86[12],	3.60[13]	4.06($^1B_{2u}/^1B_{3u}$)	-
-	4.40[13]	4.53($^1B_{2u}/^1B_{3u}$)	4.36 ($^1B_{2u}$)
4.71[9], 4.83[9]	-	-	4.85 ($^1B_{3u}$)
5.27[11], 5.39[9]	5.30[13]	5.16($^1B_{2u}/^1B_{3u}$)	5.48 ($^1B_{2u}/^1B_{3u}$)
5.82[9]	-	5.88($^1B_{3u}$)	5.85($^1B_{3u}$)
6.32[9]	6.00[13]	6.33($^1B_{2u}/^1B_{3u}$)	6.19 ($^1B_{2u}/^1B_{3u}$)
6.50[9]	-	-	6.45 ($^1B_{2u}$)
6.63[9]	6.60[13]	6.85($^1B_{2u}/^1B_{3u}$)	6.73 ($^1B_{3u}$)
[a]TDDFT method, [c]DFT(Kohan-Sham) method			

Table S8. Comparison of calculated peak locations in the optical absorption spectra of RGM-42, with the experimental results on t-butyl saturated teranthene. No other theoretical results are available for this molecule. Our calculations were performed using the PPP-MRSDCI approach, employing both the screened and the standard parameters. All results are in eV units.

Experiment[20]	This work	
	Screened	Standard
1.17,1.21,	-	-
1.41, 1.57	1.86 ($^1B_{2u}$)	2.04($^1B_{2u}$)
2.96	-	-
3.19	3.56 ($^1B_{2u}/^1B_{3u}$)	-
3.87	3.96($^1B_{2u}$)	3.80($^1B_{3u}$)
-	-	4.02 ($^1B_{2u}$)
-	4.15($^1B_{2u}$)	4.21 ($^1B_{3u}$)
-	4.53($^1B_{3u}$)	4.52($^1B_{2u}$)

Table S9. Comparison of calculated peak locations in the optical absorption spectra of RGM-50, with the experimental, and other theoretical results, for pentarylene. The calculations were performed using the PPP-MRSDCI approach, employing both the screened and the standard parameters. All results are in eV units.

Experiments	Theory (others)	This work	
		Screened	Standard
1.66[7,11]	1.40[10], 1.51^c/1.54^a[13], 1.60[14], 1.97[16]	1.72 ($^1B_{2u}$)	1.98 ($^1B_{2u}$)
3.28[11], 3.45[11]	-	3.39 ($^1B_{2u}$)	-
-	4.0[13]	3.91($^1B_{2u}/^1B_{3u}$)	3.84 ($^1B_{2u}$)
-	-	4.21($^1B_{3u}$)	
4.62[11]	4.5[13]	-	4.71 ($^1B_{3u}$)
4.80[11]	5.2[13]	4.97($^1B_{2u}/^1B_{3u}$)	5.12($^1B_{2u}/^1B_{3u}$)
5.29[11]	-	5.41($^1B_{3u}$)	5.34 ($^1B_{3u}$)
-	-	5.73($^1B_{3u}$)	5.62 ($^1B_{2u}/^1B_{3u}$)
-	6.1[13]	5.95($^1B_{3u}$)	5.99 ($^1B_{3u}$)
-	-	6.23($^1B_{2u}$)	6.41 ($^1B_{2u}/^1B_{3u}$)
-	7.4[13]	-	6.96($^1B_{3u}$)
^{a} TDDFT method, ^{c}DFT(Kohan-Sham) method			

Table S10. The calculated peak locations in the optical absorption spectra of RGM-54. No other theoretical and experimental results are available for this molecule. Our calculations were performed using the PPP-MRSDCI approach, employing both the screened and the standard parameters. All results are in eV units.

This work	
Screened	Standard
1.63 ($^1B_{2u}$)	2.09 ($^1B_{2u}$)
2.56 ($^1B_{3u}$)	-
2.83 ($^1B_{2u}$)	-
3.09 ($^1B_{2u}$)	3.20($^1B_{2u}/^1B_{3u}$)
3.71 ($^1B_{2u}/^1B_{3u}$)	3.69 ($^1B_{2u}$)
3.95 ($^1B_{3u}$)	3.98 ($^1B_{2u}/^1B_{3u}$)
4.15($^1B_{2u}$)	4.22($^1B_{3u}$)
4.31($^1B_{2u}$)	4.60 ($^1B_{3u}$)
-	4.97 ($^1B_{3u}$)
5.14 ($^1B_{2u}$)	5.14($^1B_{2u}$)
5.40 ($^1B_{3u}$)	5.41 ($^1B_{3u}$)
5.60 ($^1B_{3u}$)	-
5.82 ($^1B_{3u}$)	5.97 ($^1B_{2u}/^1B_{3u}$)
-	6.22 ($^1B_{2u}$)
-	6.56 ($^1B_{2u}/^1B_{3u}$)

Table S11. Comparison of the calculated peak locations in the optical absorption spectra of RGM-56, with the experimental results of Konishi et al.[20] No other previous theoretical calculations of absorption spectrum exist for this molecule. The calculations were performed using the PPP-MRSDCI approach, employing both the screened and the standard parameters. All results are in eV units.

Experiment[20]	This work	
	Screened	Standard
1.35, 2.01, 2.10,	1.50 ($^1B_{2u}$)	1.91($^1B_{2u}$)
2.20, 2.27, 2.32	-	-
-	2.79 ($^1B_{2u}/^1B_{3u}$)	-
3.21	-	3.35($^1B_{2u}/^1B_{3u}$)
3.46	3.61($^1B_{2u}/^1B_{3u}$)	-
-	3.92($^1B_{3u}$)	3.87($^1B_{2u}$)

Detailed Information About the Excited States

In the following tables, we present detailed information about the excitation energies, dominant many-body wave-functions, and transition dipole matrix elements of excited states with respect to the ground state (1^1A_g). The coefficient of charge conjugate of a given configuration is abbreviated as '$c.c.$' while the sign (+/-) preceding '$c.c.$' indicates that the two coefficients have (same/opposite) signs. Symbol H denotes HOMO, while L denotes LUMO. Similarly $H-n$ and $L+m$ denote n-th orbital below HOMO, and m-th orbtial above LUMO, respectively. The symbol $|H \rightarrow L\rangle$ denotes a singly excited configuration obtained by promoting one electron from HOMO to LUMO, with respect to the closed-shell Hartree-Fock reference state. Similarly, one can deduce the meaning of other singly-excited configurations such as $|H-1 \rightarrow L+2\rangle$ *etc.* The symbol $|H \rightarrow L; H-1 \rightarrow L+1\rangle$ denotes a doubly-excited configuration obtained by exciting two electrons from the Hartree-Fock reference state, one from HOMO to LUMO, the other one from HOMO-1 to LUMO+1. Nature of other doubly-excited configurations can also be deduced, similarly.

Table S12. Excited states giving rise to peaks in the singlet linear optical absorption spectrum of RGM-28, computed employing the MRSDCI approach, along with the screened parameters in the PPP model Hamiltonian.

Peak	State	E (eV)	Transition Dipole (Å)	Dominant Contributing Configurations
I_y	1^1B_{2u}	2.00	1.5360	$\|H \rightarrow L\rangle$ (0.8548)
				$\|H \rightarrow L; H-2 \rightarrow L\rangle - c.c.$ (0.0772)
II_x	3^1B_{3u}	3.87	1.7616	$\|H \rightarrow L+3\rangle + c.c.$ (0.5731)
				$\|H \rightarrow L; H-4 \rightarrow L\rangle + c.c.$ (0.1326)
III_x	5^1B_{3u}	4.19	1.3240	$\|H \rightarrow L; H \rightarrow L+1\rangle + c.c.$ (0.4853)
				$\|H \rightarrow L+6\rangle + c.c.$ (0.1959)
IV_y	7^1B_{2u}	4.49	1.5286	$\|H-1 \rightarrow L+1\rangle$ (0.7867)
				$\|H-2 \rightarrow L; H-2 \rightarrow L; H-1 \rightarrow L+1\rangle$(0.2613)
$V_{x\&y}$	11^1B_{3u}	5.16	0.6575	$\|H-2 \rightarrow L+1\rangle - c.c.$(0.4371)
				$\|H \rightarrow L; H \rightarrow L+1\rangle + c.c.$ (0.2663)
	11^1B_{2u}	5.28	0.2400	$\|H-4 \rightarrow L+1\rangle + c.c.$ (0.5264)
				$\|H \rightarrow L; H-1 \rightarrow L+3\rangle + c.c.$ (0.1417)
VI_x	15^1B_{3u}	5.63	1.7420	$\|H-2 \rightarrow L+4\rangle - c.c.$ (0.5536)
				$\|H \rightarrow L; H-2 \rightarrow L+3\rangle - c.c.$ (0.1496)
VII_y	20^1B_{2u}	6.05	1.0110	$\|H-3 \rightarrow L+3\rangle$(0.4650)
				$\|H-4 \rightarrow L+4\rangle$(0.3576)
VIII_y	28^1B_{2u}	6.41	0.7615	$\|H-5 \rightarrow L+5\rangle$(0.5875)
				$\|H \rightarrow L; H-5 \rightarrow L+5\rangle$ (0.1877)
IX_x	35^1B_{3u}	6.83	0.3116	$\|H-3 \rightarrow L+7\rangle + c.c.$(0.2755)
				$\|H \rightarrow L; H-10 \rightarrow L\rangle - c.c.$(0.2310)
$X_{x\&y}$	42^1B_{3u}	7.01	0.4071	$\|H-3 \rightarrow L; H-2 \rightarrow L\rangle - c.c.$(0.2491)
				$\|H \rightarrow L; H-3 \rightarrow L+2\rangle - c.c.$(0.2072)
	41^1B_{2u}	7.05	0.3347	$\|H-6 \rightarrow L+3\rangle + c.c.$ (0.2771)
				$\|H \rightarrow L; H-6 \rightarrow L+1\rangle + c.c.$ (0.2136)
$XI_{x\&y}$	51^1B_{2u}	7.42	0.1846	$\|H-5 \rightarrow L+7\rangle - c.c.$ (0.2568)
				$\|H-1 \rightarrow L+1; H-2 \rightarrow L\rangle - c.c.$ (0.1918)
	55^1B_{3u}	7.45	0.3834	$\|H \rightarrow L; H-7 \rightarrow L+1\rangle - c.c.$(0.2306)
				$\|H \rightarrow L+2; H-1 \rightarrow L+2\rangle + c.c.$(0.2209)

Table S13. Excited states giving rise to peaks in the singlet linear optical absorption spectrum of RGM-28, computed employing the MRSDCI approach, along with the standard parameters in the PPP model Hamiltonian.

Peak	State	E (eV)	Transition Dipole (Å)	Dominant Contributing Configurations
I_y	1^1B_{2u}	2.21	1.4389	$\|H \rightarrow L\rangle$ (0.8536)
				$\|H \rightarrow L, H \rightarrow L+2\rangle + c.c.(0.0849)$
$II_{x\&y}$	4^1B_{2u}	4.06	0.3971	$\|H \rightarrow L+5\rangle - c.c.(0.5552)$
				$\|H-1 \rightarrow L+1\rangle$ (0.2901)
	3^1B_{3u}	4.22	1.1237	$\|H \rightarrow L+3\rangle - c.c.(0.4573)$
				$\|H \rightarrow L, H-4 \rightarrow L\rangle - c.c.(0.3029)$
$III_{x\&y}$	5^1B_{2u}	4.67	0.5575	$\|H-1 \rightarrow L+1\rangle$ (0.5095)
				$\|H-2 \rightarrow L+2\rangle$ (0.3126)
	5^1B_{3u}	4.69	1.2210	$\|H-6 \rightarrow L\rangle + c.c.(0.3689)$
				$\|H \rightarrow L, H-1 \rightarrow L\rangle - c.c.(0.3661)$
$IV_{x\&y}$	8^1B_{3u}	5.06	1.0076	$\|H \rightarrow L+6\rangle - c.c.(0.3732)$
				$\|H-2 \rightarrow L+1\rangle - c.c.(0.2678)$
	8^1B_{2u}	5.12	0.9628	$\|H-1 \rightarrow L+1\rangle$ (0.4325)
				$\|H \rightarrow L; H \rightarrow L+2\rangle + c.c.(0.3316)$
V_y	10^1B_{2u}	5.41	0.5798	$\|H-2 \rightarrow L+2\rangle$ (0.4009)
				$\|H-7 \rightarrow L\rangle + c.c.(0.3928)$
$VI_{x\&y}$	14^1B_{2u}	5.95	0.7520	$\|H-3 \rightarrow L+3\rangle(0.3328)$
				$\|H-4 \rightarrow L+4\rangle(0.2553)$
	15^1B_{3u}	6.17	1.9231	$\|H-2 \rightarrow L+4\rangle - c.c.(0.5124)$
				$\|H \rightarrow L; H-3 \rightarrow L+2\rangle - c.c.(0.1875)$
VII_y	23^1B_{2u}	6.80	1.2395	$\|H-5 \rightarrow L+5\rangle(0.5205)$
				$\|H-3 \rightarrow L+3\rangle(0.1825)$
$VIII_{x\&y}$	42^1B_{2u}	7.64	0.4478	$\|H \rightarrow L; H-2 \rightarrow L+5\rangle + c.c.(0.2853)$
				$\|H-2 \rightarrow L+8\rangle + c.c.(0.2637)$
	44^1B_{3u}	7.69	0.2694	$\|H-1 \rightarrow L+1; H \rightarrow L+4\rangle - c.c.(0.2086)$
				$\|H-3 \rightarrow L+7\rangle - c.c.(0.1962)$
IX_x	50^1B_{3u}	7.97	0.4189	$\|H \rightarrow L; H-1 \rightarrow L+5\rangle - c.c.(0.2043)$
				$\|H-1 \rightarrow L+1; H \rightarrow L+4\rangle - c.c.(0.1691)$
$X_{x\&y}$	58^1B_{2u}	8.18	0.2211	$\|H-3 \rightarrow L+3\rangle$ (0.2680)
				$\|H \rightarrow L+3; H \rightarrow L+4\rangle + c.c.(0.1792)$
	58^1B_{3u}	8.21	0.5030	$\|H-6 \rightarrow L+7\rangle + c.c.(0.2280)$
				$\|H \rightarrow L+3; H \rightarrow L+2\rangle + c.c.(0.1795)$

Table S14. Excited states giving rise to peaks in the singlet linear optical absorption spectrum of RGM-30, computed employing the MRSDCI approach, along with the screened parameters in the PPP model Hamiltonian.

Peak	State	E (eV)	Transition Dipole (Å)	Dominant Contributing Configurations
I_y	1^1B_{2u}	2.11	2.3375	$\vert H \rightarrow L\rangle$ (0.8651)
				$\vert H \rightarrow L; H-1 \rightarrow L\rangle - c.c.$ (0.0599)
II_x	5^1B_{3u}	4.07	1.4797	$\vert H-3 \rightarrow L\rangle - c.c.(0.5760)$
				$\vert H \rightarrow L; H-4 \rightarrow L\rangle + c.c.(0.1246)$
III_x	8^1B_{3u}	4.58	0.5733	$\vert H-7 \rightarrow L\rangle - c.c.$ (0.5012)
				$\vert H \rightarrow L; H \rightarrow L+2\rangle + c.c.(0.2531)$
IV_y	9^1B_{2u}	5.12	1.7943	$\vert H-2 \rightarrow L+2\rangle(0.7819)$
				$\vert H-2 \rightarrow L+2; H \rightarrow L; H \rightarrow L\rangle(0.1668)$
V_x	15^1B_{3u}	5.35	1.4599	$\vert H-4 \rightarrow L+1\rangle - c.c.(0.5163)$
				$\vert H \rightarrow L; H-1 \rightarrow L+5\rangle - c.c.$ (0.1461)
$VI_{x\&y}$	17^1B_{2u}	5.95	0.6331	$\vert H-5 \rightarrow L+5\rangle(0.5465)$
				$\vert H-3 \rightarrow L+3\rangle(0.3267)$
	21^1B_{3u}	6.08	1.1819	$\vert H-5 \rightarrow L+6\rangle - c.c.(0.3385)$
				$\vert H \rightarrow L+1; H \rightarrow L+5\rangle - c.c.$ (0.2842)
VII_x	25^1B_{3u}	6.47	0.6478	$\vert H-6 \rightarrow L+5\rangle(0.3222)$
				$\vert H \rightarrow L+1; H \rightarrow L+5\rangle - c.c.(0.2789)$
VIII_x	33^1B_{3u}	6.86	0.3873	$\vert H \rightarrow L; H \rightarrow L+9\rangle + c.c.(0.3376)$
				$\vert H-6 \rightarrow L+7\rangle + c.c.(0.2862)$
IX_y	41^1B_{2u}	7.13	0.3865	$\vert H-7 \rightarrow L+7\rangle(0.3649)$
				$\vert H \rightarrow L; H-5 \rightarrow L+2\rangle + c.c.(0.3236)$
X_y	47^1B_{2u}	7.35	0.2615	$\vert H \rightarrow L; H-5 \rightarrow L+4\rangle + c.c.(0.3426)$
				$\vert H-7 \rightarrow L+7\rangle + c.c.(0.2615)$

Table S15. Excited states giving rise to peaks in the singlet linear optical absorption spectrum of RGM-30, computed employing the MRSDCI approach, along with the standard parameters in the PPP model Hamiltonian.

Peak	State	E (eV)	Transition Dipole (Å)	Dominant Contributing Configurations
I_y	1^1B_{2u}	2.43	2.1023	$\|H \rightarrow L\rangle$ (0.8590)
				$\|H-1 \rightarrow L+1\rangle$ (0.1237)
$II_{x\&y}$	3^1B_{2u}	4.64	0.3034	$\|H \rightarrow L+6\rangle + c.c.$(0.4912)
				$\|H-1 \rightarrow L+1\rangle$ (0.3289)
	6^1B_{3u}	4.75	1.0041	$\|H \rightarrow L+3\rangle - c.c.$(0.4511)
				$\|H \rightarrow L; H-4 \rightarrow L\rangle - c.c.$(0.2387)
III_x	8^1B_{3u}	5.03	0.3272	$\|H \rightarrow L+7\rangle + c.c.$ (0.4483)
				$\|H \rightarrow L+5\rangle - c.c.$(0.2445)
$IV_{x\&y}$	8^1B_{2u}	5.51	0.8169	$\|H-2 \rightarrow L+2\rangle$(0.6622)
				$\|H-1 \rightarrow L+1\rangle$(0.2024)
	9^1B_{3u}	5.54	0.9651	$\|H-2 \rightarrow L+1\rangle + c.c.$(0.5163)
				$\|H \rightarrow L; H \rightarrow L+2\rangle + c.c.$ (0.3113)
$V_{x\&y}$	12^1B_{3u}	5.87	0.7562	$\|H \rightarrow L; H \rightarrow L+4\rangle - c.c.$ (0.3994)
				$\|H-4 \rightarrow L+1\rangle - c.c.$(0.2612)
	10^1B_{2u}	6.01	1.8870	$\|H-3 \rightarrow L+3\rangle$(0.4839)
				$\|H-4 \rightarrow L+4\rangle$(0.3714)
VI_x	16^1B_{3u}	6.24	0.7562	$\|H-1 \rightarrow L+4\rangle - c.c.$(0.4007)
				$\|H \rightarrow L; H-1 \rightarrow L+5\rangle - c.c.$ (0.2706)
$VII_{x\&y}$	18^1B_{2u}	6.66	0.5494	$\|H-5 \rightarrow L+5\rangle$(0.5177)
				$\|H-3 \rightarrow L+3\rangle$(0.3348)
	21^1B_{3u}	6.85	1.6773	$\|H-5 \rightarrow L+6\rangle - c.c.$(0.2971)
				$\|H-1 \rightarrow L; H-5 \rightarrow L\rangle - c.c.$ (0.2522)
$VIII_{x\&y}$	31^1B_{2u}	7.36	0.3006	$\|H \rightarrow L; H-2 \rightarrow L+5\rangle - c.c.$(0.2227)
				$\|H-8 \rightarrow L+1\rangle - c.c.$(0.2210)
	28^1B_{3u}	7.40	0.5732	$\|H-5 \rightarrow L+6\rangle - c.c.$(0.2448)
				$\|H \rightarrow L+6; H \rightarrow L+2\rangle + c.c.$ (0.1799)
$IX_{x\&y}$	36^1B_{3u}	7.77	0.4482	$\|H-7 \rightarrow L+7\rangle$(0.3649)
				$\|H-7 \rightarrow L+7\rangle + c.c.$(0.2615)
	41^1B_{2u}	7.80	0.2955	$\|H \rightarrow L; H-5 \rightarrow L+1\rangle - c.c.$(0.2707)
				$\|H \rightarrow L; H-1 \rightarrow L+3\rangle - c.c.$(0.2109)

Table S16. Excited states giving rise to peaks in the singlet linear optical absorption spectrum of RGM-36, computed employing the MRSDCI approach, along with the screened parameters in the PPP model.

Peak	State	E (eV)	Transition Dipole (Å)	Dominant Contributing Configurations
I_y	1^1B_{2u}	2.11	1.5754	$\|H \rightarrow L\rangle$ (0.8489)
				$\|H-1 \rightarrow L+1\rangle$ (0.0983)
II_x	3^1B_{3u}	3.63	1.5116	$\|H \rightarrow L; H \rightarrow L+1\rangle - c.c.$ (0.5303)
				$\|H \rightarrow L+6\rangle + c.c.$(0.1512)
III_y	4^1B_{2u}	3.77	1.4492	$\|H-1 \rightarrow L+1\rangle$ (0.6509)
				$\|H \rightarrow L; H \rightarrow L; H-1 \rightarrow L+1\rangle$ (0.3502)
$IV_{x\&y}$	5^1B_{2u}	3.96	0.8565	$\|H-3 \rightarrow L\rangle + c.c.$ (0.5177)
				$\|H-1 \rightarrow L+1\rangle$ (0.3355)
	5^1B_{3u}	4.07	2.5241	$\|H-4 \rightarrow L\rangle + c.c.$(0.5683)
				$\|H \rightarrow L; H-5 \rightarrow L\rangle + c.c.$ (0.1575)
V_y	7^1B_{2u}	4.35	1.0823	$\|H \rightarrow L; H-2 \rightarrow L\rangle + c.c.$ (0.5281)
				$\|H-2 \rightarrow L+2\rangle$ (0.2239)
VI_x	12^1B_{3u}	4.94	1.1461	$\|H-2 \rightarrow L+1\rangle - c.c.$ (0.5074)
				$\|H \rightarrow L; H \rightarrow L+1\rangle - c.c.$ (0.1596)
VII_y	16^1B_{2u}	5.65	0.7655	$\|H-1 \rightarrow L+7\rangle + c.c.$ (0.4220)
				$\|H-3 \rightarrow L+3\rangle$ (0.3345)
$VIII_{x\&y}$	24^1B_{3u}	5.99	1.6611	$\|H-2 \rightarrow L+5\rangle + c.c.$ (0.4889)
				$\|H \rightarrow L; H-1 \rightarrow L+3\rangle - c.c.$ (0.2647)
	21^1B_{2u}	5.99	1.1893	$\|H-3 \rightarrow L+3\rangle$ (0.6155)
				$\|H \rightarrow L; H \rightarrow L; H-3 \rightarrow L+3\rangle$ (0.3149)
IX_y	28^1B_{2u}	6.50	1.1989	$\|H-5 \rightarrow L+5\rangle$ (0.6153)
				$\|H-4 \rightarrow L+4\rangle$ (0.4676)
X_x	43^1B_{3u}	6.94	0.3140	$\|H-3 \rightarrow L+8\rangle + c.c.$ (0.3827)
				$\|H-1 \rightarrow L; H-3 \rightarrow L\rangle - c.c.$ (0.2328)
XI_x	47^1B_{3u}	7.21	0.2879	$\|H \rightarrow L; H-2 \rightarrow L+4\rangle - c.c.$ (0.2177)
				$\|H \rightarrow L; H-2 \rightarrow L+6\rangle - c.c.$ (0.1931)

Table S17. Excited states giving rise to peaks in the singlet linear optical absorption spectrum of RGM-36, computed employing the MRSDCI approach, along with the standard parameters in the PPP model.

Peak	State	E (eV)	Transition Dipole (Å)	Dominant Contributing Configurations
I_y	1^1B_{2u}	2.30	1.406716	$\|H \rightarrow L\rangle$ (0.8352)
				$\|H-1 \rightarrow L+1\rangle$ (0.1018)
II_y	4^1B_{2u}	3.87	0.913194	$\|H-1 \rightarrow L+1\rangle$ (0.4670)
				$\|H-4 \rightarrow L\rangle - c.c.$(0.4507)
$III_{x\&y}$	5^1B_{2u}	4.35	0.727174	$\|H-1 \rightarrow L+1\rangle$ (0.4166)
				$\|H \rightarrow L+4\rangle - c.c.$(0.3382)
	5^1B_{3u}	4.48	2.205762	$\|H-3 \rightarrow L\rangle - c.c.$(0.4162)
				$\|H \rightarrow L; H-1 \rightarrow L\rangle + c.c.$ (0.2513)
IV_y	7^1B_{2u}	4.88	1.213884	$\|H \rightarrow L; H \rightarrow L+2\rangle - c.c.$ (0.4285)
				$\|H-1 \rightarrow L+1\rangle$(0.3659)
V_x	9^1B_{3u}	5.09	1.125146	$\|H \rightarrow L+6\rangle + c.c.$(0.4371)
				$\|H-1 \rightarrow L+2\rangle - c.c.$(0.2737)
VI_x	13^1B_{3u}	5.60	0.436946	$\|H \rightarrow L; H-7 \rightarrow L\rangle - c.c.$ (0.4290)
				$\|H-1 \rightarrow L; H-4 \rightarrow L\rangle - c.c.$ (0.2342)
$VII_{x\&y}$	14^1B_{2u}	5.82	0.367347	$\|H-5 \rightarrow L+1\rangle - c.c.$ (0.3652)
				$\|H-8 \rightarrow L\rangle + c.c.$ (0.2564)
	14^1B_{3u}	5.91	1.162591	$\|H-2 \rightarrow L+1\rangle - c.c.$ (0.3358)
				$\|H \rightarrow L; H \rightarrow L+10\rangle + c.c.$ (0.2292)
VIII_x	22^1B_{3u}	6.57	1.624379	$\|H-5 \rightarrow L+2\rangle + c.c.$ (0.4256)
				$\|H \rightarrow L+12\rangle + c.c.$ (0.1711)
IX_y	31^1B_{2u}	7.00	1.129647	$\|H-3 \rightarrow L+3\rangle$ (0.4576)
				$\|H-4 \rightarrow L+4\rangle$ (0.2268)
X_y	50^1B_{2u}	7.78	0.466491	$\|H \rightarrow L; H-6 \rightarrow L+1\rangle - c.c.$ (0.3067)
				$\|H-11 \rightarrow L+1\rangle + c.c.$ (0.2359)

Table S18. Excited states giving rise to peaks in the singlet linear optical absorption spectrum of RGM-40, computed employing the MRSDCI approach, along with the screened parameters in the PPP model.

Peak	State	E (eV)	Transition Dipole (Å)	Dominant Contributing Configurations
I_y	1^1B_{2u}	2.02	3.1106	$\|H \rightarrow L\rangle$ (0.8557)
				$\|H \rightarrow L; H-1 \rightarrow L\rangle - c.c.$ (0.0659)
$II_{x\&y}$	4^1B_{2u}	3.97	0.7433	$\|H-1 \rightarrow L+1\rangle$(0.5418)
				$\|H \rightarrow L+3\rangle + c.c.$(0.4542)
	6^1B_{3u}	4.16	1.4209	$\|H-4 \rightarrow L\rangle + c.c.$(0.5430)
				$\|H \rightarrow L; H-5 \rightarrow L\rangle + c.c.$ (0.1380)
$III_{x\&y}$	7^1B_{2u}	4.46	0.4448	$\|H \rightarrow L; H-1 \rightarrow L\rangle - c.c.$ (0.4580)
				$\|H-1 \rightarrow L+1\rangle$ (0.2558)
	10^1B_{3u}	4.60	0.8227	$\|H-2 \rightarrow L+1\rangle + c.c.$ (0.4438)
				$\|H \rightarrow L; H \rightarrow L+2\rangle - c.c.$ (0.2781)
$IV_{x\&y}$	10^1B_{2u}	5.16	2.0518	$\|H-2 \rightarrow L+2\rangle$(0.7916)
				$\|H-2 \rightarrow L+2; H \rightarrow L; H \rightarrow L\rangle$(0.1569)
	16^1B_{3u}	5.17	1.1558	$\|H-1 \rightarrow L+5\rangle - c.c.$ (0.3482)
				$\|H \rightarrow L; H \rightarrow L+2\rangle - c.c.$ (0.2872)
V_x	25^1B_{3u}	5.88	1.3378	$\|H-6 \rightarrow L+3\rangle - c.c.$(0.3284)
				$\|H-1 \rightarrow L; H-6 \rightarrow L\rangle + c.c.$ (0.3143)
$VI_{x\&y}$	29^1B_{2u}	6.30	1.1841	$\|H-7 \rightarrow L+7\rangle$(0.5752)
				$\|H-14 \rightarrow L\rangle + c.c.$(0.2588)
	34^1B_{3u}	6.37	0.6869	$\|H-4 \rightarrow L; H \rightarrow L+1\rangle - c.c.$ (0.0682)
				$\|H-1 \rightarrow L; H-4 \rightarrow L\rangle - c.c.$ (0.1899)
$VII_{x\&y}$	49^1B_{3u}	6.89	0.3030	$\|H \rightarrow L+3; H \rightarrow L+7\rangle + c.c.$ (0.3611)
				$\|H \rightarrow L; H-7 \rightarrow L+3\rangle + c.c.$ (0.2973)
	51^1B_{2u}	7.03	0.3937	$\|H \rightarrow L; H-2 \rightarrow L+4\rangle - c.c.$(0.3445)
				$\|H \rightarrow L; H-6 \rightarrow L+2\rangle - c.c.$(0.2926)

Table S19. Excited states giving rise to peaks in the singlet linear optical absorption spectrum of RGM-40, computed employing the MRSDCI approach, along with the standard parameters in the PPP model.

Peak	State	E (eV)	Transition Dipole (Å)	Dominant Contributing Configurations
I_y	1^1B_{2u}	2.30	2.7613	$\|H \rightarrow L\rangle$ (0.8402)
				$\|H-1 \rightarrow L+1\rangle$ (0.1470)
II_y	3^1B_{2u}	4.36	0.5470	$\|H \rightarrow L+3\rangle + c.c.(0.5117)$
				$\|H-1 \rightarrow L+1\rangle$ (0.3179)
III_x	6^1B_{3u}	4.85	0.8881	$\|H-4 \rightarrow L\rangle + c.c.$ (0.3945)
				$\|H \rightarrow L; H \rightarrow L+5\rangle + c.c.(0.2458)$
$IV_{x\&y}$	10^1B_{3u}	5.41	0.8911	$\|H-1 \rightarrow L+2\rangle + c.c.(0.3678)$
				$\|H \rightarrow L; H-2 \rightarrow L\rangle - c.c.(0.2935)$
	9^1B_{2u}	5.55	1.1210	$\|H-2 \rightarrow L+2\rangle(0.6631)$
				$\|H-8 \rightarrow L+8\rangle(0.1829)$
V_x	15^1B_{3u}	5.85	0.7202	$\|H \rightarrow L; H \rightarrow L+5\rangle + c.c.(0.3695)$
				$\|H-1 \rightarrow L+5\rangle - c.c.(0.2291)$
$VI_{x\&y}$	20^1B_{3u}	6.16	0.6527	$\|H \rightarrow L+1; H-6 \rightarrow L\rangle - c.c.(0.3325)$
				$\|H-1 \rightarrow L+5\rangle - c.c.(0.2940)$
	15^1B_{2u}	6.23	1.9321	$\|H-4 \rightarrow L+4\rangle(0.3439)$
				$\|H-7 \rightarrow L+7\rangle(0.3086)$
VII_y	18^1B_{2u}	6.45	0.7057	$\|H \rightarrow L; H \rightarrow L+9\rangle + c.c.(0.2227)$
				$\|H \rightarrow L+1; H \rightarrow L+3\rangle - c.c.(0.2885)$
VIII_x	27^1B_{3u}	6.73	1.6524	$\|H-1 \rightarrow L, H-6 \rightarrow L\rangle - c.c.(0.2650)$
				$\|H-3 \rightarrow L+6\rangle + c.c.(0.2570)$
IX_y	29^1B_{2u}	7.09	0.7071	$\|H-7 \rightarrow L+7\rangle(0.2946)$
				$\|H-14 \rightarrow L\rangle - c.c.(0.2479)$
$X_{x\&y}$	42^1B_{3u}	7.56	1.4486	$\|H-7 \rightarrow L+9\rangle - c.c.(0.2612)$
				$\|H \rightarrow L+3; H \rightarrow L+7\rangle - c.c.(0.2116)$
	47^1B_{2u}	7.75	0.6221	$\|H-8 \rightarrow L+8\rangle(0.2639)$
				$\|H \rightarrow L; H-12 \rightarrow L\rangle + c.c.(0.1783)$
XI_x	57^1B_{3u}	8.14	0.5141	$\|H-1 \rightarrow L; H-1 \rightarrow L+7\rangle - c.c.(0.1957)$
				$\|H \rightarrow L; H-3 \rightarrow L+5\rangle - c.c.(0.1675)$

Table S20. Excited states giving rise to peaks in the singlet linear optical absorption spectrum of RGM-42, computed employing the MRSDCI approach, along with the screened parameters in the PPP model.

Peak	State	E (eV)	Transition Dipole (Å)	Dominant Contributing Configurations
I_y	1^1B_{2u}	1.86	2.5210	$\|H \rightarrow L\rangle$ (0.8412)
				$\|H \rightarrow L; H-2 \rightarrow L\rangle - c.c.$ (0.1094)
$II_{x\&y}$	4^1B_{3u}	3.50	1.2044	$\|H \rightarrow L; H \rightarrow L+1\rangle + c.c.$ (0.4984)
				$\|H-6 \rightarrow L\rangle + c.c.$(0.2112)
	4^1B_{2u}	3.62	1.2714	$\|H \rightarrow L; H-2 \rightarrow L\rangle - c.c.$ (0.4764)
				$\|H-2 \rightarrow L+2\rangle$ (0.2997)
III_x	7^1B_{3u}	3.96	2.1501	$\|H-3 \rightarrow L\rangle - c.c.$(0.5100)
				$\|H \rightarrow L; H-4 \rightarrow L\rangle - c.c.$ (0.4764)
IV_y	7^1B_{2u}	4.15	2.0730	$\|H-1 \rightarrow L+1\rangle$ (0.7196)
				$\|H \rightarrow L; H \rightarrow L; H-1 \rightarrow L+1\rangle$(0.3491)
V_x	11^1B_{3u}	4.53	0.5587	$\|H-2 \rightarrow L+1\rangle - c.c.$(0.4956)
				$\|H \rightarrow L; H-4 \rightarrow L\rangle + c.c.$ (0.2330)
VI_x	12^1B_{3u}	4.76	0.7880	$\|H \rightarrow L; H-4 \rightarrow L\rangle + c.c.$ (0.4333)
				$\|H \rightarrow L+3\rangle - c.c.$(0.2272)
VII_x	18^1B_{3u}	5.38	1.6773	$\|H-2 \rightarrow L+4\rangle - c.c.$(0.5205)
				$\|H \rightarrow L; H-3 \rightarrow L+2\rangle + c.c.$ (0.1605)
VIII_x	26^1B_{3u}	5.89	0.8326	$\|H \rightarrow L; H-3 \rightarrow L+2\rangle + c.c.$ (0.2690)
				$\|H \rightarrow L+2; H \rightarrow L+3\rangle + c.c.$ (0.2374)
$IX_{x\&y}$	34^1B_{2u}	6.25	1.2975	$\|H-7 \rightarrow L+7\rangle$(0.4661)
				$\|H \rightarrow L; H-7 \rightarrow L+2\rangle + c.c.$(0.2892)
	39^1B_{3u}	6.34	0.9598	$\|H \rightarrow L; H-1 \rightarrow L+7\rangle + c.c.$(0.2847)
				$\|H \rightarrow L; H-6 \rightarrow L+2\rangle - c.c.$(0.2512)

Table S21. Excited states giving rise to peaks in the singlet linear optical absorption spectrum of RGM-42, computed employing the MRSDCI approach, along with the standard parameters in the PPP model.

Peak	State	E (eV)	Transition Dipole (Å)	Dominant Contributing Configurations
I_y	1^1B_{2u}	2.04	2.2700	$\|H \rightarrow L\rangle$ (0.8368)
				$\|H \rightarrow L, H \rightarrow L+1\rangle$ $-c.c.$(0.1091)
II_x	4^1B_{3u}	3.80	0.4640	$\|H \rightarrow L, H-2 \rightarrow L\rangle$ $+c.c.$(0.3563)
				$|H \rightarrow L+6\rangle$$+c.c.$ (0.3059)
III_y	5^1B_{2u}	4.02	0.8072	$|H \rightarrow L+8\rangle$$-c.c.$ (0.3624)
				$|H \rightarrow L+5\rangle$$+c.c.$ (0.2890)
IV_x	6^1B_{3u}	4.21	1.0272	$\|H \rightarrow L+3\rangle - c.c.$(0.3949)
				$\|H \rightarrow L; H \rightarrow L+4\rangle - c.c.$(0.2569)
V_y	7^1B_{2u}	4.52	1.0353	$\|H-1 \rightarrow L+1\rangle$ (0.4140)
				$|H-5 \rightarrow L\rangle$$+c.c.$(0.3504)
$VI_{x\&y}$	9^1B_{2u}	4.76	1.6034	$\|H-2 \rightarrow L+2\rangle$(0.5386)
				$|H \rightarrow L; H \rightarrow L+1\rangle$$-c.c.$(0.2354)
	10^1B_{3u}	4.77	1.2151	$|H-7 \rightarrow L\rangle$$-c.c.$(0.3885)
				$|H \rightarrow L+3\rangle$$-c.c.$(0.2395)
$VII_{x\&y}$	12^1B_{2u}	5.33	0.4950	$\|H-1 \rightarrow L+1\rangle$ (0.4422)
				$\|H \rightarrow L; H \rightarrow L; H-1 \rightarrow L+1\rangle$(0.3336)
	14^1B_{3u}	5.47	1.1069	$|H \rightarrow L+3\rangle$$-c.c.$(0.2835)
				$|H \rightarrow L; H-4 \rightarrow L\rangle$$-c.c.$(0.2819)
VIII_x	19^1B_{3u}	6.03	1.0756	$|H-1 \rightarrow L+4\rangle$$+c.c.$(0.4085)
				$|H \rightarrow L; H-6 \rightarrow L+1\rangle$$-c.c.$(0.2487)
$IX_{x\&y}$	23^1B_{2u}	6.27	0.7868	$\|H-3 \rightarrow L+3\rangle$ (0.2978)
				$\|H-6 \rightarrow L+6\rangle$ (0.2779)
	24^1B_{3u}	6.31	1.0124	$\|H \rightarrow L; H \rightarrow L; H-1 \rightarrow L+2\rangle - c.c.$(0.2976)
				$\|H \rightarrow L; H-3 \rightarrow L+1\rangle + c.c.$(0.1875)
$X_{x\&y}$	29^1B_{2u}	6.52	0.5814	$\|H \rightarrow L+14\rangle - c.c.$(0.2337)
				$\|H \rightarrow L; H-2 \rightarrow L+6\rangle + c.c.$(0.2331)
	29^1B_{3u}	6.58	0.8128	$\|H-1 \rightarrow L; H-7 \rightarrow L\rangle + c.c.$(0.2177)
				$\|H \rightarrow L+1; H \rightarrow L+6\rangle + c.c.$(0.1815)
XI_x	34^1B_{3u}	6.84	1.2787	$\|H \rightarrow L+2; H \rightarrow L+8\rangle - c.c.$(0.2013)
				$\|H \rightarrow L; H-2 \rightarrow L+5\rangle + c.c.$(0.1815)
$XII_{x\&y}$	49^1B_{2u}	7.26	1.1191	$\|H-8 \rightarrow L+8\rangle$(0.3712)
				$\|H-3 \rightarrow L+3\rangle$(0.2236)
	48^1B_{3u}	7.34	1.0043	$|H \rightarrow L; H-1 \rightarrow L+6\rangle$$-c.c.$(0.2911)
				$\|H-5 \rightarrow L+6\rangle + c.c.$(0.2512)

Table S22. Excited states giving rise to peaks in the singlet linear optical absorption spectrum of RGM-50, computed employing the MRSDCI approach, along with the screened parameters in the PPP model.

Peak	State	E (eV)	Transition Dipole (Å)	Dominant Contributing Configurations
I_y	1^1B_{2u}	1.72	3.8432	$\lvert H \rightarrow L\rangle$ (0.8590)
				$\lvert H \rightarrow L; H \rightarrow L+1\rangle + c.c.$ (0.0678)
II_y	4^1B_{2u}	3.39	0.9517	$\lvert H-1 \rightarrow L+1\rangle$(0.5734)
				$\lvert H-2 \rightarrow L\rangle - c.c.$(0.4401)
$III_{x\&y}$	8^1B_{2u}	3.79	0.5483	$\lvert H{\rightarrow}L; H \rightarrow L+1\rangle + c.c.$ (0.4451)
				$\lvert H-1{\rightarrow}L+1\rangle$ (0.2769)
	8^1B_{3u}	4.03	1.2328	$\lvert H{\rightarrow}L+4\rangle - c.c.$ (0.3874)
				$\lvert H-8{\rightarrow}L\rangle + c.c.$ (0.2867)
IV_x	11^1B_{3u}	4.21	0.9785	$\lvert H-1 \rightarrow L+3\rangle - c.c.$(0.4115)
				$\lvert H \rightarrow L; H \rightarrow L+3\rangle - c.c.$(0.2970)
$V_{x\&y}$	20^1B_{3u}	4.90	1.1721	$\lvert H-5 \rightarrow L+1\rangle + c.c.$(0.4231)
				$\lvert H{\rightarrow}L; H-5 \rightarrow L\rangle + c.c.$ (0.2244)
	17^1B_{2u}	5.04	2.0716	$\lvert H-3 \rightarrow L+3\rangle$(0.8044)
				$\lvert H \rightarrow L; H \rightarrow L+3; H-3 \rightarrow L\rangle$ (0.1640)
VI_x	31^1B_{3u}	5.41	1.0791	$\lvert H-6 \rightarrow L; H-1 \rightarrow L\rangle + c.c.$(0.2361)
				$\lvert H \rightarrow L+1; H-6 \rightarrow L\rangle - c.c.$(0.2227)
VII_x	40^1B_{3u}	5.73	0.7423	$\lvert H-2 \rightarrow L+3\rangle + c.c.$(0.2486)
				$\lvert H-6 \rightarrow L+2\rangle - c.c.$(0.2406)
VII_x	50^1B_{3u}	5.95	0.6274	$\lvert H-6 \rightarrow L; H-1 \rightarrow L\rangle + c.c.$(0.2361)
				$\lvert H-6 \rightarrow L+2\rangle - c.c.$(0.2406)
IX_y	35^1B_{2u}	6.23	0.8094	$\lvert H-8 \rightarrow L+8\rangle$(0.6294)
				$\lvert H \rightarrow L; H-3 \rightarrow L+8\rangle - c.c.$(0.2972)

Table S23. Excited states giving rise to peaks in the singlet linear optical absorption spectrum of RGM-50, computed employing the MRSDCI approach, along with the standard parameters in the PPP model.

Peak	State	E (eV)	Transition Dipole (Å)	Dominant Contributing Configurations
I_y	1^1B_{2u}	1.98	3.4780	$\|H \rightarrow L\rangle$ (0.8368)
				$\|H-1 \rightarrow L+1\rangle$ (0.1685)
II_y	4^1B_{2u}	3.84	0.6241	$\|H-1 \rightarrow L+1\rangle$ (0.6657)
				$\|H-2 \rightarrow L\rangle + c.c.$ (0.2916)
III_x	8^1B_{3u}	4.71	0.8499	$\|H-4 \rightarrow L\rangle + c.c.$ (0.3818)
				$\|H \rightarrow L, H \rightarrow L+5\rangle - c.c.$(0.2012)
$IV_{x\&y}$	12^1B_{3u}	5.10	1.0796	$\|H-3 \rightarrow L+1\rangle - c.c.$(0.3378)
				$\|H \rightarrow L; H \rightarrow L+3\rangle - c.c.$(0.2762)
	9^1B_{2u}	5.14	0.5471	$\|H \rightarrow L; H \rightarrow L+1\rangle + c.c.$(0.3645)
				$\|H-1 \rightarrow L+1\rangle$ (0.2258)
V_x	15^1B_{3u}	5.34	0.6970	$\|H-8 \rightarrow L+1\rangle - c.c.$(0.3202)
				$\|H \rightarrow L; H-8 \rightarrow L\rangle - c.c.$(0.2821)
$VI_{x\&y}$	13^1B_{2u}	5.61	1.1140	$\|H-3 \rightarrow L+3\rangle$(0.5543)
				$\|H-1 \rightarrow L+1\rangle$(0.2919)
	18^1B_{3u}	5.64	0.7958	$\|H \rightarrow L; H-5 \rightarrow L\rangle - c.c.$(0.3125)
				$\|H-1 \rightarrow L+5\rangle - c.c.$(0.2359)
VII_x	25^1B_{3u}	5.99	0.6481	$\|H-1 \rightarrow L+12\rangle - c.c.$(0.2424)
				$\|H-14 \rightarrow L\rangle + c.c.$(0.2348)
$VIII_{x\&y}$	23^1B_{2u}	6.38	1.8981	$\|H-4 \rightarrow L+4\rangle$ (0.3313)
				$\|H-9 \rightarrow L+9\rangle$(0.3307)
	32^1B_{3u}	6.45	1.4693	$\|H \rightarrow L+6; H \rightarrow L+1\rangle - c.c.$(0.2247)
				$\|H \rightarrow L; H-1 \rightarrow L+10\rangle - c.c.$(0.1985)
IX_x	42^1B_{3u}	6.96	0.3205	$\|H \rightarrow L; H-2 \rightarrow L+3\rangle - c.c.$(0.2100)
				$\|H \rightarrow L; H-1 \rightarrow L+4\rangle - c.c.$(0.2050)

Table S24. Excited states giving rise to peaks in the singlet linear optical absorption spectrum of RGM-54, computed employing the MRSDCI approach, along with the screened parameters in the PPP model.

Peak	State	E (eV)	Transition Dipole (Å)	Dominant Contributing Configurations
I_y	1^1B_{2u}	1.63	1.4845	$\|H \rightarrow L\rangle$ (0.8330)
				$\|H-1 \rightarrow L+1\rangle$ (0.1286)
II_x	2^1B_{3u}	2.56	2.2031	$\|H \rightarrow L; H \rightarrow L+1\rangle + c.c.$ (0.4984)
				$\|H-4 \rightarrow L\rangle - c.c.$(0.1687)
III_y	4^1B_{2u}	2.83	2.0444	$\|H-1 \rightarrow L+1\rangle$ (0.6233)
				$\|H \rightarrow L; H \rightarrow L; H-1 \rightarrow L+1\rangle$ (0.5382)
IV_y	5^1B_{2u}	3.09	2.1949	$\|H \rightarrow L; H-2 \rightarrow L\rangle - c.c.$ (0.5340)
				$\|H-1 \rightarrow L+1\rangle$ (0.1943)
$V_{x\&y}$	9^1B_{3u}	3.66	1.9235	$\|H \rightarrow L+6\rangle + c.c.$(0.3849)
				$\|H \rightarrow L; H \rightarrow L+7\rangle - c.c.$ (0.3397)
	8^1B_{2u}	3.76	0.4426	$\|H-5 \rightarrow L\rangle + c.c.$(0.5363)
				$\|H \rightarrow L; H \rightarrow L; H-1 \rightarrow L+1\rangle$ (0.1882)
VI_x	12^1B_{3u}	3.95	1.6583	$\|H-1 \rightarrow L+2\rangle + c.c.$ (0.5033)
				$\|H-8 \rightarrow L\rangle - c.c.$(0.1636)
VII_y	12^1B_{2u}	4.15	0.8667	$\|H-2 \rightarrow L+2\rangle$(0.6638)
				$\|H \rightarrow L; H \rightarrow L; H-2 \rightarrow L+2\rangle$ (0.4977)
VIII_x	17^1B_{3u}	4.31	0.7625	$\|H \rightarrow L; H-7 \rightarrow L\rangle - c.c.$ (0.4137)
				$\|H \rightarrow L+6\rangle + c.c.$ (0.2776)
IX_y	29^1B_{2u}	5.14	1.2230	$\|H-3 \rightarrow L+3\rangle$(0.4616)
				$\|H \rightarrow L; H \rightarrow L; H-3 \rightarrow L+3\rangle$ (0.3651)
X_x	39^1B_{3u}	5.40	1.4639	$\|H-2 \rightarrow L+7\rangle + c.c.$ (0.3768)
				$\|H \rightarrow L; H-2 \rightarrow L+6\rangle - c.c.$ (0.3183)
XI_x	43^1B_{3u}	5.60	0.6826	$\|H \rightarrow L; H-2 \rightarrow L+6\rangle - c.c.$ (0.4033)
				$\|H \rightarrow L; H-2 \rightarrow L+8\rangle - c.c.$ (0.2236)
XII_x	48^1B_{3u}	5.82	0.6093	$\|H-11 \rightarrow L+1\rangle - c.c.$ (0.3254)
				$\|H \rightarrow L+17\rangle + c.c.$ (0.2814)

Table S25. Excited states giving rise to peaks in the singlet linear optical absorption spectrum of RGM-54, computed employing the MRSDCI approach, along with the standard parameters in the PPP model.

Peak	State	E (eV)	Transition Dipole (Å)	Dominant Contributing Configurations
I_y	1^1B_{2u}	2.09	1.4091	$\|H \rightarrow L\rangle$ (0.8149)
				$\|H-1 \rightarrow L+1\rangle$ (0.1651)
$II_{x\&y}$	2^1B_{3u}	3.15	1.6323	$\|H \rightarrow L; H \rightarrow L+1\rangle - c.c.$ (0.4840)
				$\|H-4 \rightarrow L\rangle - c.c.$(0.2369)
	4^1B_{2u}	3.25	1.2639	$\|H-1 \rightarrow L+1\rangle$ (0.4851)
				$\|H \rightarrow L; H \rightarrow L; H-1 \rightarrow L+1\rangle$ (0.4840)
III_y	5^1B_{2u}	3.69	1.1580	$\|H \rightarrow L+3\rangle + c.c.$ (0.4030)
				$\|H \rightarrow L; H-2 \rightarrow L\rangle + c.c.$ (0.3073)
$IV_{x\&y}$	6^1B_{2u}	3.91	2.0968	$\|H-1 \rightarrow L+1\rangle$ (0.3495)
				$\|H-3 \rightarrow L\rangle + c.c.$(0.3201)
	7^1B_{3u}	4.04	1.5506	$\|H-4 \rightarrow L\rangle + c.c.$(0.3500)
				$\|H \rightarrow L; H \rightarrow L+7\rangle + c.c.$(0.3310)
V_x	9^1B_{3u}	4.22	1.1430	$\|H \rightarrow L+4\rangle - c.c.$(0.3000)
				$\|H \rightarrow L+8\rangle + c.c.$(0.2912)
VI_x	11^1B_{3u}	4.64	0.6269	$\|H \rightarrow L+8\rangle + c.c.$(0.4151)
				$\|H-5 \rightarrow L\rangle - c.c.$(0.3113)
VII_x	14^1B_{3u}	4.97	1.0338	$\|H-1 \rightarrow L+2\rangle - c.c.$ (0.4195)
				$\|H \rightarrow L; H \rightarrow L+7\rangle + c.c.$ (0.1741)
VIII_y	14^1B_{2u}	5.14	0.9203	$\|H-1 \rightarrow L+9\rangle - c.c.$ (0.3905)
				$\|H \rightarrow L; H \rightarrow L; H-9 \rightarrow L+1\rangle - c.c.$(0.2940)
IX_x	19^1B_{3u}	5.41	0.7882	$\|H \rightarrow L; H-3 \rightarrow L+1\rangle - c.c.$ (0.2563)
				$\|H \rightarrow L; H \rightarrow L+7\rangle + c.c.$ (0.2369)
$X_{x\&y}$	26^1B_{2u}	5.91	0.6193	$\|H \rightarrow L; H-4 \rightarrow L+1\rangle - c.c.$ (0.2488)
				$\|H-4 \rightarrow L+4\rangle$ (0.2293)
	28^1B_{3u}	6.02	0.4809	$\|H-6 \rightarrow L; H-1 \rightarrow L\rangle + c.c.$ (0.3414)
				$\|H \rightarrow L+2; H \rightarrow L+4\rangle - c.c.$ (0.2686)
XI_y	33^1B_{2u}	6.22	1.0196	$\|H \rightarrow L; H \rightarrow L; H-3 \rightarrow L+3\rangle$ (0.3850)
				$\|H-3 \rightarrow L+3\rangle$ (0.3640)
$XII_{x\&y}$	38^1B_{3u}	6.51	1.6007	$\|H-2 \rightarrow L+7\rangle + c.c.$ (0.2954)
				$\|H \rightarrow L+2; H \rightarrow L+4\rangle - c.c.$ (0.2515)
	45^1B_{2u}	6.61	0.7244	$\|H-3 \rightarrow L+3\rangle$ (0.2293)
				$\|H-1 \rightarrow L+1; H \rightarrow L+2\rangle + c.c.$ (0.2139)

Table S26. Excited states giving rise to peaks in the singlet linear optical absorption spectrum of RGM-56, computed employing the MRSDCI approach, along with the screened parameters in the PPP model.

Peak	State	E (eV)	Transition Dipole (Å)	Dominant Contributing Configurations
I_y	1^1B_{2u}	1.50	2.7694	$\|H \rightarrow L\rangle$ (0.8452)
				$\|H \rightarrow L; H \rightarrow L+1\rangle - c.c.$ (0.1424)
$II_{x\&y}$	3^1B_{2u}	2.76	2.2125	$\|H \rightarrow L; H \rightarrow L+1\rangle - c.c.$ (0.5010)
				$\|H-1 \rightarrow L\rangle + c.c.$(0.2824)
	2^1B_{3u}	2.82	1.6228	$\|H \rightarrow L; H-2 \rightarrow L\rangle - c.c.$ (0.5242)
				$\|H-4 \rightarrow L\rangle - c.c.$(0.2594)
$III_{x\&y}$	8^1B_{2u}	3.61	2.3531	$\|H-2 \rightarrow L+2\rangle$ (0.6437)
				$\|H \rightarrow L; H \rightarrow L; H-2 \rightarrow L+2\rangle$ (0.4553)
	8^1B_{3u}	3.61	1.7934	$\|H \rightarrow L+6\rangle - c.c.$(0.4248)
				$\|H \rightarrow L; H-7 \rightarrow L\rangle - c.c.$ (0.3464)
IV_x	11^1B_{3u}	3.92	1.0255	$\|H-1 \rightarrow L+2\rangle + c.c.$ (0.4865)
				$\|H \rightarrow L; H \rightarrow L+9\rangle + c.c.$ (0.1941)
$V_{x\&y}$	11^1B_{2u}	4.32	0.3844	$\|H \rightarrow L; H \rightarrow L+10\rangle - c.c.$ (0.3907)
				$\|H \rightarrow L; H \rightarrow L; H-1 \rightarrow L+1\rangle$ (0.3841)
	16^1B_{3u}	4.35	0.4780	$\|H \rightarrow L; H \rightarrow L+7\rangle - c.c.$ (0.3674)
				$\|H \rightarrow L+6\rangle + c.c.$(0.2270)
$VI_{x\&y}$	23^1B_{2u}	4.97	0.5917	$\|H \rightarrow L; H-4 \rightarrow L+2\rangle + c.c.$ (0.4252)
				$\|H \rightarrow L+1; H-2 \rightarrow L+2\rangle - c.c.$ (0.2884)
	27^1B_{3u}	5.03	1.7568	$\|H-7 \rightarrow L+1\rangle + c.c.$(0.2270)
				$\|H \rightarrow L; H-1 \rightarrow L+6\rangle - c.c.$ (0.2162)
VII_y	30^1B_{2u}	5.22	0.4515	$\|H \rightarrow L; H-2 \rightarrow L+8\rangle + c.c.$ (0.4065)
				$\|H-4 \rightarrow L+4\rangle$(0.2276)
VIII_x	49^1B_{3u}	5.88	0.5430	$\|H \rightarrow L; H-5 \rightarrow L+2\rangle + c.c.$ (0.4801)
				$\|H-7 \rightarrow L+1\rangle + c.c.$(0.2123)

Table S27. Excited states giving rise to peaks in the singlet linear optical absorption spectrum of RGM-56, computed employing the MRSDCI approach, along with the standard parameters in the PPP model.

Peak	State	E (eV)	Transition Dipole (Å)	Dominant Contributing Configurations
I_y	1^1B_{2u}	1.91	2.8248	$\|H \to L\rangle$ (0.8337)
				$\|H \to L; H \to L+1\rangle - c.c.$ (0.1496)
$II_{x\&y}$	3^1B_{2u}	3.32	1.1646	$\|H \to L; H \to L+1\rangle - c.c.$ (0.3783)
				$\|H-1 \to L+1\rangle$ (0.3586)
	3^1B_{3u}	3.38	0.9728	$\|H \to L; H-2 \to L\rangle + c.c.$ (0.4057)
				$\|H \to L+4\rangle + c.c.$ (0.3460)
III_y	5^1B_{2u}	3.87	1.2282	$\|H \to L+7\rangle + c.c.$ (0.4874)
				$\|H-2 \to L+2\rangle$ (0.3066)
$IV_{x\&y}$	7^1B_{2u}	4.09	0.6195	$\|H-1 \to L+1\rangle$ (0.4561)
				$\|H \to L+3\rangle - c.c.$(0.3551)
	7^1B_{3u}	4.15	1.3814	$\|H \to L+8\rangle - c.c.$(0.3505)
				$\|H \to L; H \to L+6\rangle - c.c.$(0.2916)
V_y	9^1B_{2u}	4.39	1.7225	$\|H-2 \to L+2\rangle$(0.4006)
				$\|H \to L; H \to L+1\rangle - c.c.$(0.2769)
$VI_{x\&y}$	10^1B_{3u}	4.51	1.0202	$\|H-5 \to L\rangle - c.c.$(0.3087)
				$\|H \to L+8\rangle - c.c.$(0.2574)
	11^1B_{2u}	4.63	1.4111	$\|H-2 \to L+2\rangle$(0.3934)
				$\|H-1 \to L+1\rangle$(0.3614)
$VII_{x\&y}$	15^1B_{3u}	5.09	0.6254	$\|H-2 \to L+1\rangle - c.c.$ (0.3123)
				$\|H \to L; H-9 \to L\rangle - c.c.$ (0.2352)
	13^1B_{2u}	5.20	0.4511	$\|H \to L; H \to L; H-1 \to L+1\rangle$ (0.4445)
				$\|H \to L; H \to L; H-2 \to L+2\rangle$ (0.3259)
VIII_x	17^1B_{3u}	5.38	0.7487	$\|H \to L; H-11 \to L\rangle - c.c.$ (0.3117)
				$\|H-5 \to L\rangle - c.c.$(0.2132)
$IX_{x\&y}$	24^1B_{3u}	5.94	1.1856	$\|H-6 \to L+1\rangle + c.c.$(0.3017)
				$\|H-4 \to L; H-1 \to L\rangle - c.c.$ (0.2391)
	24^1B_{2u}	5.99	0.3409	$\|H-3 \to L+3\rangle$(0.3329)
				$\|H \to L+1; H \to L+7\rangle - c.c.$ (0.2871)
X_x	28^1B_{3u}	6.11	0.7549	$\|H \to L; H-3 \to L+2\rangle - c.c.$ (0.3810)
				$\|H \to L+1; H-1 \to L+2\rangle + c.c.$ (0.1974)
$XI_{x\&y}$	29^1B_{2u}	6.29	0.4309	$\|H \to L; H-2 \to L+4\rangle + c.c.$ (0.2998)
				$\|H \to L+1; H \to L+7\rangle - c.c.$ (0.2614)
	34^1B_{3u}	6.39	1.2890	$\|H-3 \to L+4\rangle - c.c.$ (0.3028)
				$\|H \to L; H-3 \to L+2\rangle - c.c.$ (0.2642)
$XII_{x\&y}$	41^1B_{3u}	6.67	0.7789	$\|H-8 \to L; H-1 \to L\rangle + c.c.$ (0.2255)
				$\|H \to L; H-1 \to L+8\rangle - c.c.$ (0.2050)
	38^1B_{2u}	6.77	0.7244	$\|H-4 \to L+4\rangle$ (0.3114)
				$\|H \to L; H \to L; H-4 \to L+4\rangle$ (0.2800)

References

1. Konishi, A., Hirao, Y., Matsumoto, K., Kurata, H. & Kubo, T. Facile synthesis and lateral π-expansion of bisanthenes. *Chem. Lett.* **42**, 592–594, DOI: 10.1246/cl.130153 (2013). https://doi.org/10.1246/cl.130153.
2. Clar, E. & Schmidt, W. Correlations between photoelectron and ultraviolet absorption spectra of polycyclic hydrocarbons. the perylene, coronene and bisanthene series. *Tetrahedron* **33**, 2093–2097 (1977).
3. Malloci, G. See the UV-Vis absorption spectrum of bisanthene posted at http://www.dsf.unica.it/~gmalloci/pahs/bisanthene/bisanthene.html.
4. Arabei, S. & Pavich, T. Spectral-luminescent properties and photoinduced transformations of bisanthene and bisanthenequinone. *J. Appl. Spectrosc.* **67**, 236–244 (2000).

5. Parac, M. & Grimme, S. A tddft study of the lowest excitation energies of polycyclic aromatic hydrocarbons. *Chem. physics* **292**, 11–21 (2003).
6. Kummer, S. *et al.* Absorption, excitation, and emission spectroscopy of terrylene in p-terphenyl: Bulk measurements and single molecule studies. *The J. Chem. Phys.* **107**, 7673–7684 (1997).
7. Baumgarten, M., Koch, K.-H. & Muellen, K. Spin density distribution and electronic structure of radical anions of ladder-type oligorylenes. *J. Am. Chem. Soc.* **116**, 7341–7348 (1994).
8. Biktchantaev, I., Samartsev, V. & Sepiol, J. Perylene and terrylene in rare gas matrixes: spectroscopic and computational studies of inclusion sites. *J. luminescence* **98**, 265–272 (2002).
9. Halasinski, T. M. *et al.* Electronic absorption spectra of neutral perylene (c20h12), terrylene (c30h16), and quaterrylene (c40h20) and their positive and negative ions: Ne matrix-isolation spectroscopy and time-dependent density functional theory calculations. *The J. Phys. Chem. A* **107**, 3660–3669 (2003).
10. Viruela-Martín, R., Viruela-Martín, P. M. & Orti, E. Theoretical determination of the geometric and electronic structures of oligorylenes and poli (peri-naphthalene). *The J. chemical physics* **97**, 8470–8480 (1992).
11. Koch, K.-H. & Müllen, K. Polyarylenes and poly (arylenevinylene) s, v. synthesis of tetraalkyl-substituted oligo (1, 4-naphthylene) s and cyclization to soluble oligo (peri-naphthylene) s2). *Chemische Berichte* **124**, 2091–2100 (1991).
12. Ruiterkamp, R. *et al.* Spectroscopy of large pahs-laboratory studies and comparison to the diffuse interstellar bands. *Astron. & Astrophys.* **390**, 1153–1170 (2002).
13. Malloci, G., Cappellini, G., Mulas, G. & Mattoni, A. Electronic and optical properties of families of polycyclic aromatic hydrocarbons: A systematic (time-dependent) density functional theory study. *Chem. Phys.* **384**, 19–27 (2011).
14. Minami, T., Ito, S. & Nakano, M. Theoretical study of singlet fission in oligorylenes. *The journal physical chemistry letters* **3**, 2719–2723 (2012).
15. Clar, E. & Schmidt, W. Correlations between photoelectron and ultraviolet absorption spectra of polycyclic hydrocarbons: the terrylene and peropyrene series. *Tetrahedron* **34**, 3219–3224 (1978).
16. Karabunarliev, S., Gherghel, L., Koch, K.-H. & Baumgarten, M. Structure and optical absorption of oligorylenes upon doping. *Chem. physics* **189**, 53–65 (1994).
17. Malloci, G. See the uv-vis absorption spectrum of tetrabenzocoronene posted at http://www.dsf.unica.it/ gmalloci/pahs/tetrabenzocoronene/tetrabenzocoronene.html.
18. Former, C., Becker, S., Grimsdale, A. C. & Müllen, K. Cyclodehydrogenation of poly (perylene) to poly (quaterrylene): Toward poly (peri-naphthalene). *Macromolecules* **35**, 1576–1582 (2002).
19. Gudipati, M. S. & Allamandola, L. J. Double ionization of quaterrylene (c40h20) in water-ice at 20 k with lyr (121.6 nm) radiation. *life* **5**, 10 (2006).
20. Konishi, A. *et al.* Anthenes: Model systems for understanding the edge state of graphene nanoribbons. *Pure Appl. Chem.* **86**, 497–505 (2014).
